# Surface Study of Fe_3_O_4_ Nanoparticles Functionalized With Biocompatible Adsorbed Molecules

**DOI:** 10.3389/fchem.2019.00642

**Published:** 2019-10-04

**Authors:** Beata Lesiak, N. Rangam, P. Jiricek, I. Gordeev, J. Tóth, L. Kövér, M. Mohai, P. Borowicz

**Affiliations:** ^1^Institute of Physical Chemistry, Polish Academy of Sciences, Warsaw, Poland; ^2^Institute of Physics, Academy of Sciences of the Czech Republic, Prague, Czechia; ^3^Institute for Nuclear Research, Hungarian Academy of Sciences, Debrecen, Hungary; ^4^Research Centre for Natural Sciences, Institute of Materials and Environmental Chemistry, Hungarian Academy of Sciences, Budapest, Hungary

**Keywords:** ferrimagnetic magnetite (Fe_3_O_4_) magnetic nanoparticles, biocompatible acid functionalization, DLS, FTIR-S, TGA/DSC, XPS, REELS, HeLa cells

## Abstract

Surfaces of iron oxide of ferrimagnetic magnetite (Fe_3_O_4_) nanoparticles (MNPs) prepared by Massart's method and their functionalized form (f-MNPs) with succinic acid, L-arginine, oxalic acid, citric acid, and glutamic acid were studied by dynamic light scattering (DLS), Fourier transform infrared spectroscopy (FTIR-S), UV-vis, thermogravimetric analysis (TGA)/differential scanning calorimetry (DSC), X-ray photoelectron spectroscopy (XPS), and reflection electron energy loss spectroscopy (REELS). The XPS analysis of elements and their chemical states at the surface of MNPs and f-MNPs revealed differences in chemical bonding of atoms, content of carbon–oxygen groups, iron oxide forms, iron oxide magnetic properties, adsorbed molecules, surface coverage, and overlayer thickness, whereas the Auger parameters (derived from XPS and Auger spectra) and elastic and inelastic scattering probabilities of electrons on atoms and valence band electrons (derived from REELS spectra) indicated modification of surface charge redistribution, electronic, and optical properties. These modified properties of f-MNPs influenced their biological properties. The surfaces biocompatible for L929 cells showed various cytotoxicity for HeLa cells (10.8–5.3% of cell death), the highest for MNPs functionalized with oxalic acid. The samples exhibiting the largest efficiency possessed smaller surface coverage and thickness of adsorbed molecules layers, the highest content of oxygen and carbon–oxygen functionalizing groups, the highest ratio of lattice O^2−^ and OH^−^ to C sp^2^ hybridizations on MNP surface, the highest ratio of adsorbed O^−^ and OH^−^ to C sp^2^ hybridizations on adsorbed molecule layers, the closest electronic and optical properties to Fe_3_O_4_, and the lowest degree of admolecule polymerization. This high cytotoxicity was attributed to interaction of cells with a surface, where increased content of oxygen groups, adsorbed O^−^, and OH^−^ may play the role of additional adsorption and catalytic sites and a large content of adsorbed molecule layers of carboxylic groups facilitating Fenton reaction kinetics leading to cell damage.

## Introduction

The iron oxide nanoparticles, i.e., ferrimagnetic maghemite (γ-Fe_2_O_3_) with Fe^3+^ vacancies and ferrimagnetic magnetite (Fe_3_O_4_ ≡ FeO•Fe_2_O_3_) with Fe^2+^ and Fe^3+^ vacancies, have already been applied in the field of medicine due to their biocompatibility, biodegradability, and possibility to tailor magnetic behavior (Sangaiya and Jayaprakash, 2018), where the change of nanoparticle size, morphology, agglomeration, magnetic, and electronic properties influences the biological effect (Liu et al., 2016). Although magnetic targeting iron nanoparticles serve as platforms for attaching drugs like, e.g., doxorubicin (DOX), they were also applied in a tumor therapy, which resulted in a hyperthermia and oxidative stress leading to tumor cell damage (Rangam et al., 2017; Petran et al., 2018; Sangaiya and Jayaprakash, 2018). Enhancement of antitumor effect was obtained by functionalization of nanoparticles by a conventional DOX drug (Liu et al., 2016; Rangam et al., 2017) and/or doping with rare metals (Petran et al., 2018). Additional functionalization of iron nanoparticles may lead to enhancement of their biocompatibility, colloidal stability, and enlargement of number of groups, through which the required antitumor effect can be obtained.

The cytotoxicity of Fe_3_O_4_ MNPs coated with a wide variety of biocompatible admolecules has been recently extensively studied (Gupta and Gupta, 2005; Kim et al., 2009, 2010; Tomitaka et al., 2012; Mahdavi et al., 2013; Sahu et al., 2015; Taghavi et al., 2016; Hu et al., 2017; Linh et al., 2018). These several studies showed that cytotoxicity depends on the type of the investigated cells, type of biocompatible adlayer on MNPs, MNP size, concentration of MNPs, pH of solution, and time of incubation. The functionalization of MNPs with biocompatible molecules like polyethylene glycol, pluronic acid, and others providing a better biocompatibility may also affect the cytotoxicity due to modification of physical and chemical properties of the material, its interaction with biological cells, and ability of forming reactive oxygen species (ROS). The coating of Fe_3_O_4_ MNPs may provide a positive charge, which facilitates interaction of a specimen with negatively charged cell membrane (Taghavi et al., 2016). For the concentration range of 0.012–0.1 mg mL^−1^, the generation of ROS by Fe_3_O_4_ nanoparticles was found to be smaller in comparison to Pt and PEGylated mesoporous iron–platinum–iron(II, III) (FePt-Fe_3_O_4_) nanoparticles (Sahu et al., 2015). Recently, it has been reported that Fe_3_O_4_ MNPs exhibited no cytotoxicity for HeLa cells during incubation time from 24 to 72 h, whereas coated MNPs showed the cytotoxicity after 72 h (Linh et al., 2018). No effect of Fe_3_O_4_ MNPs on cytotoxicity for HeLa cells was observed for incubation time of 24 h elsewhere (Gupta and Gupta, 2005; Kim et al., 2009, 2010; Tomitaka et al., 2012).

Functionalized nanoparticles of Fe_3_O_4_ iron oxide prepared by Massart's method (Massart, 1981; Runowski and Lis, 2016) (f-MNPs) using functionalization with succinic acid [(CH_2_)_2_(CO_2_H)_2_], L-arginine (C_6_H_14_N_4_), oxalic acid (C_2_H_2_O_4_), citric acid (C_6_H_8_O_7_), and glutamic acid (C_5_H_9_O_4_N) showed similar biocompatibility on fibroblasts (Rangam et al., 2017). Their average efficiency on HeLa cell treatment (% of HeLa cell deaths) in an f-MNP concentration range of 3.125–100 μg mL^−1^ and incubation time of 24 h decreased in the following order: 10.8% (oxalic acid), 10.7% (succinic acid), 9.2% (glutamic acid), 7.5% (citric acid) and 5.3% (L-arginine) (Rangam et al., 2017). The respective f-MNPs loaded with DOX showed about six times higher efficiency on HeLa cell therapy decreasing in different order, i.e., 64.2% (oxalic acid), 55.2% (L-arginine), 42.8% (glutamic acid), 42.2% (succinic acid), and 32.7% (citric acid), which is attributed to DOX adsorption and modified surface properties (Rangam et al., 2017).

The reason for the different cytotoxicity of Fe_3_O_4_ MNPs functionalized with oxalic, succinic, glutamic acids, and L-arginine for HeLa cells was investigated by X-ray photoelectron spectroscopy (XPS), X-ray excited Auger electron spectroscopy (XAES), and reflected electron loss spectroscopy (REELS) revealing the chemical groups at the surface and modification of surface electronic properties. These studies were supported by dynamic light scattering (DLS), Fourier transform infrared spectroscopy (FTIR-S), UV-vis, thermogravimetric analysis (TGA), and differential scanning calorimetry (DSC).

## Experimental

### Samples

Details on synthesis of nanoparticles of iron oxide (Fe_3_O_4_) by Massart's method (MNPs) (Massart, 1981; Runowski and Lis, 2016) and their functionalization at a temperature of 70°C-80°C at pH ca. 6–7 for 30 min with succinic acid [(CH_2_)_2_(CO_2_H)_2_], L-arginine (C_6_H_14_N_4_), oxalic acid (C_2_H_2_O_4_), citric acid (C_6_H_8_O_7_), and glutamic acid (C_5_H_9_O_4_N) (samples denoted as I, II, III, IV, and V, respectively) were described elsewhere (Rangam et al., 2017). The structural formulae of functionalizing adsorbed molecules are shown in Supplementary Figure S1. The prepared samples' (Fe_3_O_4_ MNPs and Fe_3_O_4_ f-MNPs I–V) chemical, structural, and magnetic properties were characterized previously by energy dispersive X-ray spectroscopy (EDX), scanning electron microscopy (SEM), transmission electron microscopy (TEM), X-ray diffraction (XRD), and vibrating sample magnetometer (Rangam et al., 2017).

### DLS, FTIR-S, UV-vis, and TGA/DSC Apparatuses

The DLS measurements were carried out using Brookhaven Instruments Particle Size Analyzer 90+ to determine nanoparticles' hydrodynamic diameter (*D*_H_), polydispersity index (PDI), and zeta potential. The measurements were performed in a water suspension of concentration of 0.01 mg mL^−1^ at pH ca. 6.

The FTIR spectra were recorded in Fourier spectrophotometer Vertex 80 V (Bruker Inc., USA) in a configuration of attenuated total reflectance (ATR) at a pressure below 5 hPa, which reduces negative factors like carbon dioxide and water. In order to obtain a high spectral resolution and signal-to-noise ratio, the following apparatus conditions were applied during the measurement: spectral resolution of 2 cm^−1^ and number of scans of 1,024.

The UV-vis spectra were recorded in deionized water solution by a Shimazu UV-2401 spectrophotometer.

The TGS/DSC data were recorded using Mettler Toledo TGA/DSC 3+ apparatus in nitrogen flow in a temperature range from room temperature (RT) to 800°C at a heating rate of 10°C min^−1^.

### XPS Spectrometer

The XPS spectra of Fe_3_O_4_ MNPs and Fe_3_O_4_ f-MNPs I–V were measured in an ultra-high vacuum (UHV) AXIS Supra photoelectron spectrometer (Kratos Analytical, UK). The incidence angle of the monochrome Al K_α_ radiation (1 mm^2^ irradiation area, 300 × 700 μm analyzed area) was set to 54.4° and the photoelectron emission angle was α_out_ = 0°, with respect to the surface normal. The hemispherical electron energy analyzer operated in the constant analyzer energy (CAE) mode at an analyzer pass energy of *E*_p_ = 80 eV (survey spectra) and *E*_p_ = 10 eV (high-resolution detailed spectra). The data acquisition was performed using ESCApe Kratos software. The samples were investigated without any UHV pretreatment. The binding energies (BE) of all the spectra were referenced to BE of 284.4 eV of C 1s line.

The REELS measurements proceeded in an ultra-high-vacuum (UHV) chamber using the ESA-31 electron spectrometer (home-made) (Kövér et al., 1992). The spectrometer is equipped with a hemispherical electron energy analyzer of high energy resolution, an electron gun (LEG62-VG Microtech), a home-made X-ray excitation source (Al Kα X-rays hν = 1486.67 eV), and an Ar^+^ ion source of AG21 (VG Scientific). The REELS spectra were measured at fixed retardation ratio (FRR) mode using the retardation ratio of *k* = 41. The electron beam parameters were as follows: a primary electron energy of 4 keV, a beam current of about 11.5 nA measured with a Faraday cup, and electron incidence and emission angles of 50° and 0° with respect to the surface normal of the specimen, respectively.

## Results and Discussion

### DLS, FTIR-S, UV-vis, and TGA/DSC

The investigated Fe_3_O_4_ MNPs and Fe_3_O_4_ f-MNPs I–V exhibit various values of *D*_H_, PDI, and zeta potential. The values of *D*_H_ vary in a range of 217.9–871.2 nm (III < V < I < Fe_3_O_4_ < IV < II). Larger values of *D*_H_ in comparison to SEM values (Rangam et al., 2017) would suggest polymeric coating formed from adsorbed molecules on Fe_3_O_4_ MNPs. The values of polydispersity are in a range of 0.039–0.853 (IV < I < Fe_3_O_4_ < II < V < III) indicating different agglomeration/aggregation of nanoparticles in a solution. The value of zeta potential at pH ca. 6 is at a range of −0.53 to 0.83 mV (I < II < IV < V < Fe_3_O_4_ < III).

**Figure 1:**
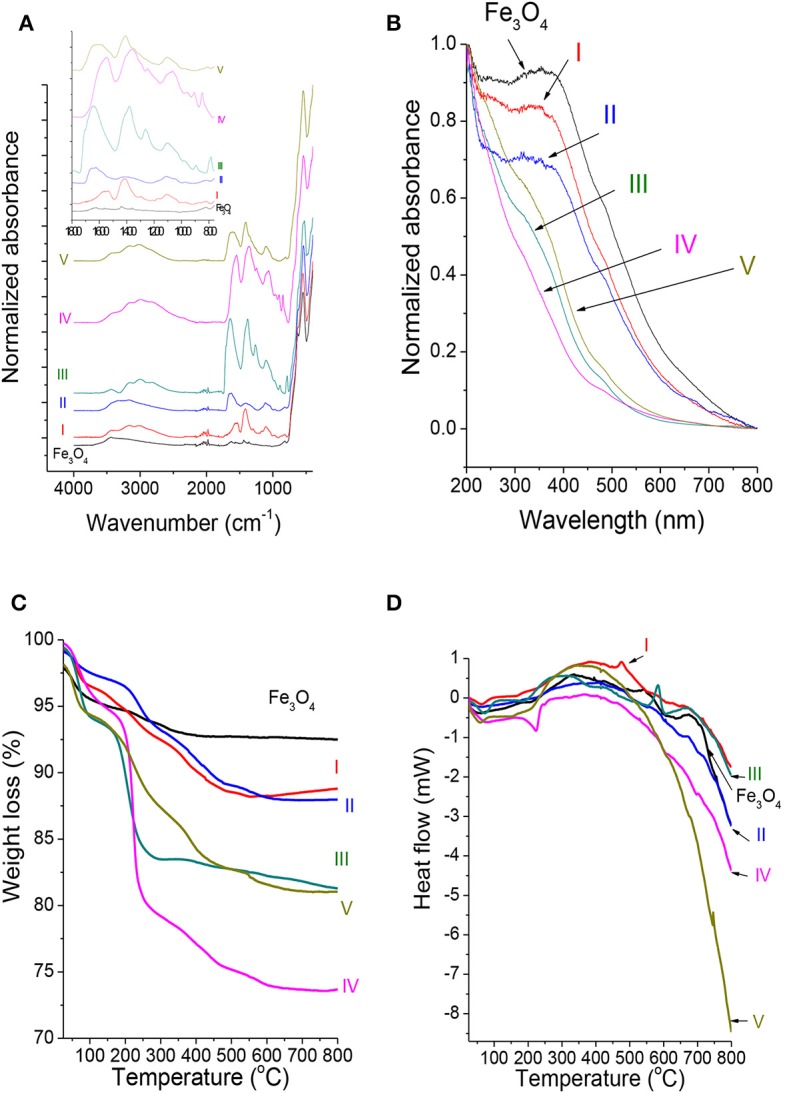
**(A)** The ATR-FTIR, **(B)** UV-vis spectra, and **(C)** TGA and **(D)** DSC curves of the investigated Fe_3_O_4_ MNPs and Fe_3_O_4_ f-MNPs I–V.

The ATR-FTIR spectra of Fe_3_O_4_ MNPs and f-MNPs I–V are shown in Figure 1A. All spectra show the characteristic peak of iron oxides, i.e., Fe-O at 548 cm^−1^. The literature reports this peak at 580 cm^−1^ (Wei et al., 2012; Asgari et al., 2014; Bordbar et al., 2014). However, these reported data result from measurements in a polar environment of KBr pellet, which may shift the peak position. The spectrum of Fe_3_O_4_ MNPs exhibits modes typical for organic groups in regions of 760–1800 cm^−1^, about 2,000 cm^−1^, and 2,500–3,600 cm^−1^. The spectra of Fe_3_O_4_ MNPs I–V samples confirm the presence of adsorbed molecule layers. All the spectra were normalized to the intensity of the Fe–O peak at 548 cm^−1^ in order to compare the intensity of peaks at different spectra regions for various adsorbed molecules. The FTIR spectra can be divided into the following regions: 760–1,180 cm^−1^, 1,180–1,480 cm^−1^, 1,480–1,800 cm^−1^, region about 2,000 cm^−1^, and 2,500–3,600 cm^−1^. In the region of 760–1,180 cm^−1^, C–C stretching (strong) and C–N stretching (medium) exist (Infrared Spectroscopy-MSU Chemistry, 2013). This region encloses the complex skeleton modes involving few vibration local modes due to modification of length of the bonds and angles between the bonds. Such vibration modes have been previously observed for L-arginine (Kumar and Rai, 2010) and glutamic acid (Sengupta and Krimm, 1985). For Fe_3_O_4_ f-MNPs II and V, dominating signal should result from C–C stretching. In the region of 1,180–1,480 cm^−1^, the functionalized MNP modes characteristic for COOH group exist, i.e., stretching C–O mode (medium strong) (Silverstein et al., 1981; Infrared Spectroscopy-MSU Chemistry, 2013), bending C–O–H mode (medium), and bending C–H mode (Silverstein et al., 1981). For the investigated admolecules, the literature reports symmetric stretching COOH^−^ mode for succinic acid (Krishnan et al., 2007); CH_3_ symmetric bending mode for L-arginine (Kumar and Rai, 2010); stretching C–O mode for oxalic acid (Muthuselvi et al., 2016); scissoring C–O–H, CH_2_, CH_3_ modes, wagging CH_2_, and CH_3_ modes for citric acid (Bichara et al., 2011); and different types of bending C–H and stretching C–O modes for glutamic acid (Sengupta and Krimm, 1985). In the region of 1,480–1,800 cm^−1^, C = O stretching mode (strong) is dominating (Silverstein et al., 1981; Infrared Spectroscopy-MSU Chemistry, 2013). For sample MNPs III, this mode is shifted to higher frequencies, and among all the samples, this mode resembles the structure of the COOH group in oxalic acid. For sample MNPs I and IV, this mode is shifted to lower frequencies, i.e., to 1,550 cm^−1^. The structure of sample MNPs III is more rigid than the structure of sample MNPs I and MNPs IV. The interaction of COOH groups in f-MNPs III with Fe_3_O_4_ is weak. This is shown in bending of a mode at about 1,705 cm^−1^ typical for the COOH group interacting weakly with Fe_3_O_4_ MNPs. The main mode maximum at about 1,645 cm^−1^ is attributed to the C = O mode of stronger interaction with MNPs. For sample MNPs I and IV, their less rigid structure allows for attraction of all COOH groups to Fe_3_O_4_, which results in a larger frequency shift of C = O mode to lower values. For sample MNPs II and V, the mode in a range of 1,490–1,705 cm^−1^, there is an overlap of stretching C = O mode (strong) (Silverstein et al., 1981; Infrared Spectroscopy-MSU Chemistry, 2013) and bending N–H mode (medium/medium-strong) typical for amine and amide (Silverstein et al., 1981; Infrared Spectroscopy-MSU Chemistry, 2013). Contribution of bending N–H mode and interaction of COOH with Fe_3_O_4_ result in a mode position and shape, where, for smaller frequencies, the slope responsible for bending N–H mode is smaller than that for stretching C = O mode. The region at about 2,000 cm^−1^ consists of weak modes responsible for combination and overtones of basic modes. The region of 2,500–3,600 cm^−1^ encloses stretching O–H modes (strong and wide depending on the environment), stretching C–H modes (generally strong), and stretching N–H mode typical for amine groups (sample MNPs II and V) (Silverstein et al., 1981; Infrared Spectroscopy-MSU Chemistry, 2013). Since the intensity of N–H stretching mode in this region is weak, the main components are stretching O–H and C–H modes with contributions depending on the sample.

The UV-vis spectra of Fe_3_O_4_ MNPs (Figure 1B) indicate the absorption band between 400 and 420 nm characteristic for Fe_3_O_4_ nanoparticles as reported elsewhere (Bahadur et al., 2017) and at about 300 nm reported previously for Fe_2_O_3_ (Bian et al., 2017). Less intense absorption bands with intensity decreasing in the order of I > II > III ≈ V > IV and shifted to smaller wavelengths exhibit Fe_3_O_4_ f-MNPs I–V samples due to adsorbed molecules. The shift may be attributed to adsorbed molecules, where L-arginine absorption band at 226–278 nm has been reported (Kumar and Rai, 2010).

The TGA curves (Figure 1C, Supplementary Figure S2) indicate various weight loss for every investigated sample in different temperature regions. The temperature range of 30–150°C is characteristic for a loss of residual water and other contaminations, as well as for physically adsorbed molecules. In the temperature range of 150–600°C, carboxyl, hydroxyl, carbonyl, and nitrogen groups of chemically adsorbed molecules undergo decomposition (Lesiak et al., 2009; Stobinski et al., 2010, 2012; Linh et al., 2018), contributing to this weight loss, whereas higher temperatures are more characteristic for phase transformations. The weight loss for all the investigated samples in the first range varies from 2.3 to 5.4% and is larger for oxalic (5.4%) and citric (4.6%) acids indicating either higher surface hydrophilicity and/or physical adsorption. The weight loss in the second region is 2.8% for Fe_3_O_4_ MNPs, 8 and 8.5% for L-arginine and succinic acid, 10.5 and 10.9% for oxalic and glutamic acid, and 21.4% for citric acid. This weight loss between 10.5 and 21.4% indicates citric, oxalic, and glutamic acid dissolution in Fe_3_O_4_, where dissolution in oxalic acid was reported elsewhere (Panias et al., 1996). Thermal stability decreases in the following order: Fe_3_O_4_ MNPs > II (f-L-arginine) > I (f-succininc acid) > III (f-oxalic acid) > V (f-glutamic acid) > IV (f-citric acid).

The DSC curves (Figure 1D, Supplementary Figure S2) show endothermic and exothermic peaks indicating, respectively, heat absorption and release. The endothermic peaks are attributed to phase transitions, reduction, and most decomposition reactions, whereas exothermic peaks are related with oxidation, decomposition reactions, and crystallization. The first endothermic peaks visible for all the samples at 50.5–71.3°C can be attributed to desorption and/or evaporation of water and is related with the weight loss from 1.9% for Fe_3_O_4_ MNPs to 5.4% for f-MNPs I–V. The endothermic peaks in a range of 190–224°C can be attributed to desorption and/or decomposition of carboxylic groups and are related with the weight loss of 1.7–15.7%. This peak maximum temperature shift, i.e., Fe_3_O_4_ MNPs < I ≈ III < IV ≈ V < II, indicates increasing binding energy between MNPs and carboxylic groups from functionalizing molecules. The other broad exothermic peaks in a range of 320–420°C (Fe_3_O_4_ MNPs < I < V < II < IV) with a weight loss of 1.1–5.7% (Fe_3_O_4_ MNPs < I < V < II < IV) may be attributed to hydroxyl, carbonyl, and nitrogen group decomposition (Lesiak et al., 2009; Stobinski et al., 2010, 2012; Linh et al., 2018). The narrow exothermic peak at 475°C for sample I (f-succininc acid) with a weight loss of 1.3% and at 583°C for sample III (f-oxalic acid) with a weight loss of 2.2% may be related to decomposition of carbonyl groups forming a stronger bond with MNPs (Lesiak et al., 2009; Stobinski et al., 2010, 2012). The above results indicate different thermal decomposition, confirming the results by FTIR-S indicating various adsorption behaviors of molecules via MNP surface interaction.

### Quantitative Surface Analysis

**Figure 2:**
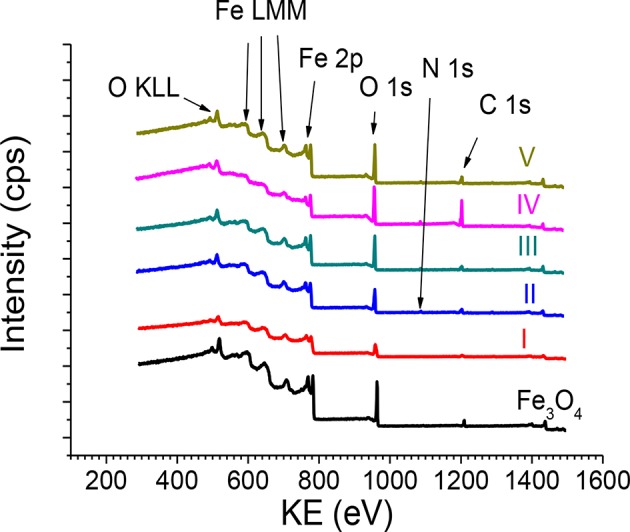
The survey XPS spectra recorded from Fe_3_O_4_ MNPs and Fe_3_O_4_ f-MNPs I–V.

The survey XPS spectra (Figure 2) showed Fe, C, O, and N and contaminations of S and Cl at the surface. The quantitative surface analysis was performed using the peak areas (Fe 2p, C 1s, O 1s, N 1s, S 2p, and Cl 2p) after Tougaard background subtraction (Tougaard, 1999–2001) using the XPS MultiQuant software (Mohai, 1999-2001, 2004) considering a homogeneous surface distribution of elements, Scofield subshell photoionization cross sections (Scofield, 1976), and correction for analyzer transmission function and electron elastic scattering. The atomic composition of samples Fe_3_O_4_ and I–V is listed in Supplementary Table S1. The Fe_3_O_4_ MNPs show contamination of only Cl. The N from functionalizing adsorbed molecules was present in samples II and V, whereas samples III and IV indicated contamination of N. Otherwise, all f-MNPs show contamination of Cl and sample IV contamination of S. Contaminations of Cl and S result from precursors used in the Massart's synthesis of Fe_3_O_4_ MNPs, whereas contamination of N from ammonia and N gas flow conditions applied in the above mentioned synthesis.

**Figure 3:**
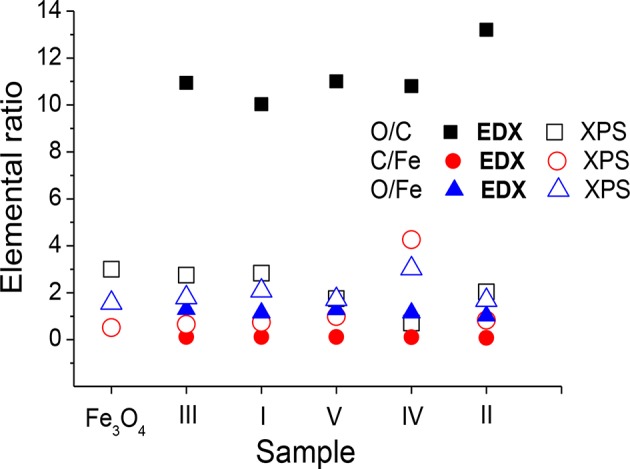
Comparison of ratios of elemental contents at the surface (XPS) and in the bulk (EDX) (Rangam et al., 2017) of Fe_3_O_4_ and Fe_3_O_4_ f-MNPs I–V. Sample f-MNPs I–V are listed in the order of decreasing cytotoxicity.

Comparison of elemental ratios at the surface resulting from XPS to those in the bulk resulting from EDX, published elsewhere (Rangam et al., 2017), is shown in Figure 3. The ratios of C/Fe and O/Fe atomic contents at the surface (XPS) are larger than the respective ratios in the bulk (EDX), indicating remarkably larger carbon and oxygen content after functionalization, which result from the formation of a carbon–oxygen layer at the surface of Fe_3_O_4_ f-MNPs. The ratio of O/C atomic content being smaller at the surface than in the bulk indicates oxygen deficiency of functionalizing surface layers in comparison to the bulk Fe_3_O_4_ MNPs. Differences in these ratio values for MNPs and f-MNPs denoted as I–V confirm various adsorbed molecules layer of different surface coverages.

### Chemical State of Carbon, Oxygen, and Nitrogen

Fitting of C 1s, O 1s, and N 1s spectra was performed after Tougaard background subtraction using the XPSPeakfit41 software (Kwok, 2000). The fitting of C 1s, O 1s, and N 1s spectra was focused on determination of the chemical forms of functionalizing overlayers and the Fe_3_O_4_ MNP substrate. The expected C chemical forms at the surface of Fe_3_O_4_ MNPs are carbon atoms forming sp^2^ and/or sp^3^ hybridizations and sp^2^ and/or sp^3^ carbon bonded to oxygen groups like carbonyl (C = O), hydroxyl (C–OH), and carboxyl (C–OOH) resulting from oxygen adsorption, whereas functionalization of the Fe_3_O_4_ MNP substrate is expected to provide increased number of carbon forms resulting from the chemical treatment with biocompatible molecules I–V, which consist mainly of C sp^2^/sp^3^ and carboxylic bonds (Supplementary Figure S1). These carbon oxygen bonds resulting from oxidation of carbon layer on Fe_3_O_4_ MNPs and the MNP additional functionalization, i.e., C = O, C–OH, and C–OOH, are expected to be present in the O 1s spectrum. However, the O 1s spectrum should also provide information on oxidized forms of Fe. Previously reported experimental and theoretical results (Butenko et al., 2005; Shim et al., 2012; Wagner et al., 2012; Fujimoto et al., 2016; Lesiak et al., 2018) provided and compiled the values of binding energy (BE) for C 1s and O 1s spectra typical for these carbon–oxygen groups for different carbon nanomaterials. Similarly, different oxidized forms of Fe due to iron treatment with oxygen and water were reported previously including the respective BE values (Grosvenor et al., 2004a,b), as well as carbon–nitrogen chemical forms (Wagner et al., 2012).

**Figure 4:**
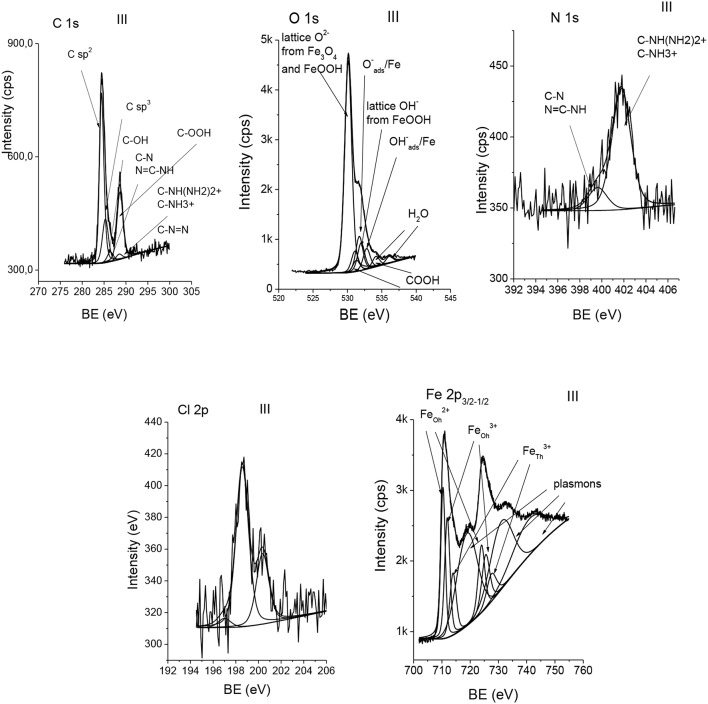
The XPS C 1s, O 1s, N 1s, Cl 2p, and Fe 2p spectra fitted to different chemical forms for exemplary Fe_3_O_4_ f-MNPs III.

**Figure 5:**
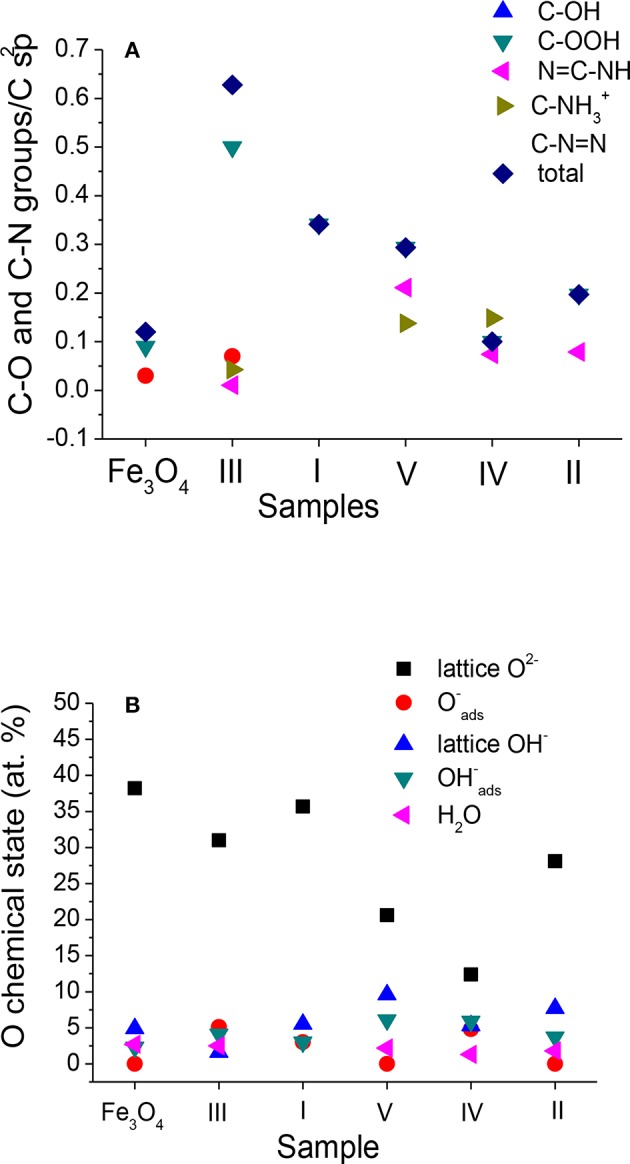
Contents of **(A)** carbon–oxygen and carbon–nitrogen groups resulting from fitting of C 1s spectra normalized to C sp^2^ and **(B)** oxygen chemical states resulting from fitting of O 1s spectra in Fe_3_O_4_ MNPs and Fe_3_O_4_ f-MNPs I–V. Sample f-MNPs I–V are listed in the order of decreasing cytotoxicity.

The resulting C 1s, O 1s, and N 1s spectra for sample III are shown in Figure 4, whereas the spectra recorded and fitted for all the samples are presented in Supplementary Figures S3A–C. The atomic contents of carbon, oxygen, and nitrogen chemical states resulting from C 1s, O 1s, and N 1s spectra fitting are listed in Supplementary Tables S2A–C. Supplementary Tables S2A–C also contains the respective BE values for C 1s, O 1s, and N 1s electrons characterizing the chemical states of the components of the adsorbed molecule layers and the Fe_3_O_4_ substrate. At the surface of Fe_3_O_4_ MNPs, the C–OH and C–OOH bonds are present, whereas f-MNPs I–V surfaces exhibit a large amount of C–OOH bonds (Supplementary Table S2A) due to functionalizing adsorbed molecules consisting profoundly of carboxylic bonds (Supplementary Figure S1). A small amount of C–OH bonds at the surface of sample III results probably from decomposition of carboxylic groups due to X-ray damage. The O 1s spectrum (Supplementary Table S2B) indicates the same amount of C–OOH groups resulting from adsorbed molecules and iron oxide forms interpreted as the lattice O^2−^ and adsorbed O^−^ from Fe_3_O_4_ and/or FeOOH and lattice OH^−^ and adsorbed OH^−^ from FeOOH, where adsorbed forms refer to those confirmed by the angular resolved measurement forms at the outer surface (Grosvenor et al., 2004a,b) and finally water adsorbed at the surface. Although, O^−^ and OH^−^ species would adsorb as atomic or molecular form, they receive the negative charge from tunneling electrons from the metal to the surface, as it has been suggested to occur during oxidation of Fe. Therefore, the notation of adsorbed O^−^ and OH^−^ includes a partial negative charge, which allows their BE values to be close to BEs for the lattice O^2−^ and OH^−^. At the surface of Fe_3_O_4_ MNPs and their I–V functionalized MNPs, the largest amount of lattice O^2−^ is observed, then lattice OH^−^, and then adsorbed OH^−^ and O^−^ forms and water. The comparison of the atomic contents of carbon and oxygen chemical states (Supplementary Tables S2A,B) for Fe_3_O_4_ MNPs and f-MNPs I–V in the order of decreasing cytotoxicity on HeLa cells is shown in Figures 5A,B, respectively.

### Chemical State of Fe

The stoichiometric magnetite Fe_3_O_4_ of cubic close packed oxygen sublattice can be alternatively expressed as FeO•Fe_2_O_3_. Therefore, it consists of iron ions Fe^2+^ and Fe^3+^ occupying the tetrahedral (denoted as Th) and octahedral (denoted as Oh) interstices of cubic spinel type structure. The ideal Fe_3_O_4_ atomic ratio of Fe^2+^:Fe^3+^ is 1:2, and precisely the ratio of Fe_Oh_^2+^:Fe_Oh_^3+^:Fe_Th_^3+^ is 1:1:1. The fitting of Fe 2p spectra accounted for Fe^2+^ and Fe^3+^ octahedral and Fe^3+^ tetrahedral spectra component parameters like Fe 2p_3/2_ BE and FWHM values reported in the literature (Yamashita and Hayes, 2008; Poulin et al., 2010; Eltouny and Ariya, 2014; Herng et al., 2015; Liu et al., 2016; Li et al., 2018; Petran et al., 2018). Exemplary Fe 2p spectra fitting results are shown in Figure 4, whereas all the fitted spectra are shown in Supplementary Figure S4. Table 1 provides the parameters of the fitted Fe 2p_3/2_ spectra recorded for Fe_3_O_4_ MNPs and f-MNPs I–V. For Fe_3_O_4_ MNPs, the obtained values of BE for Fe^2+^ octahedral and Fe^3+^ tetrahedral and octahedral components, as well as intensity ratios of Fe^3+^ and Fe^2+^ tetrahedral and octahedral components equal to 1:1:1, are in agreement with those reported previously (Poulin et al., 2010), confirming Fe_3_O_4_ MNPs (Table 1). The temperature functionalization of Fe_3_O_4_ surface by biocompatible admolecules (samples I–V) at 70–80°C modifies the stoichiometry of Fe_3_O_4_, i.e., FeO•Fe_2_O_3_, leading to changes of ratio of Fe^2+^ octahedral and Fe^3+^ tetrahedral and octahedral components, their BE values, and separation between the octahedral Fe^2+^ component and plasmon loss of the octahedral Fe^2+^ component (ΔFe_Oh_^2+^) of the Fe 2 p_3/2_ spectra, which are also reflected in magnetic properties. Consistently increasing values of BE due to functionalization, values of ratio of Fe^2+^ and Fe^3+^ components, and values of separation between Fe 2p_3/2_ octahedral 2+ component and values of separation between plasmon loss of Fe 2p_3/2_ octahedral 2+ component (ΔFe_Oh_^2+^) ranging from 8.11 to 8.77 eV (Table 1) indicate surface oxidation. Such separation values (ΔFe_Oh_^2+^), i.e., from 8.0 to 8.5 eV, were observed for α- and γ-FeOOH and Fe_2_O_3_ (Grosvenor et al., 2004c).

**Table 1:** The percentage of Fe^2+^/ Fe^3+^ octahedral (Oh) and Fe^3+^ tetrahedral (Th) chemical states including BE values of Fe 2p_3/2_ components; ratio of separation between Fe 2p_3/2_ octahedral 2+ component and plasmon loss; ratios of Fe_Oh_^2+^, Fe_Oh_^3+^, and Fe_Th_^3+^ components; ratio of plasmon loss to the total area and separation of Fe_Oh_^2+^ components from plasmon loss in Fe 2p_3/2_ spectra recorded from Fe_3_O_4_ and Fe_3_O_4_ f-MNPs I–V.

**Sample**	**Fe chemical state**						**Fe_**Oh**_^**2+**^/ Fe_**Oh**_^**3+**^ + Fe_**Th**_^**3+**^**	**Fe_**Oh**_^**2+**^:Fe_**Oh**_^**3+**^:Fe_**Th**_^**3+**^**	**A_**Plas**_/ A_**Total**_**	**ΔFe_**Oh**_^**2+**^ -plasmon**
	**Fe**_****Oh****_^****2+****^		**Fe**_****Oh****_^****3+****^		**Fe**_****Th****_^****3+****^					
	**(%)**	**BE (eV)**	**(%)**	**BE (eV)**	**(%)**	**BE (eV)**				
Fe_3_O_4_	33	710.0	33	711.4	34	713.3	0.49	1:1:1	0.5844	8.77
III	39	710.4	38	711.9	23	713.9	0.64	1:0.97:0.59	0.5857	8.52
I	30	710.8	35	712.2	35	714.0	0.43	0.86:1:1	0.5007	8.90
V	34.5	710.5	34.5	711.9	31.0	713.8	0.53	1:1:0.90	0.5855	8.32
IV	40	710.0	34	711.4	26	713.3	0.67	1:0.85:0.65	0.5986	8.11
II	37.0	710.5	39.0	712.0	24.0	714.1	0.59	0.95:1:0.62	0.5815	8.49

*Sample f-MNPs I–V are listed in the order of decreasing cytotoxicity*.

**Figure 6:**
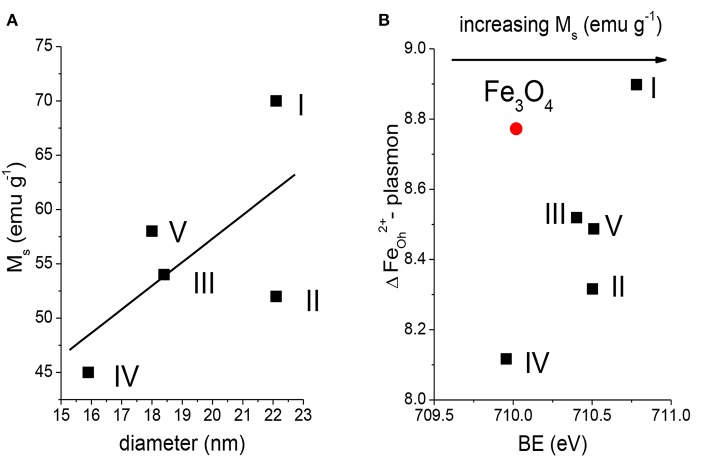
Dependence of **(A)** Fe_3_O_4_ f-MNPs I–V diameters determined from XRD (Rangam et al., 2017) as a function of saturation magnetization. **(B)** BE of Fe 2p 2+ octahedral component on separation between Fe 2p_3/2_ 2+ octahedral component and plasmon loss in Fe 2p_3/2_ spectra recorded from Fe_3_O_4_ MNPs and Fe_3_O_4_ f-MNPs I–V.

As reported previously (Rangam et al., 2017), the value of saturation magnetization, *M*_s_, for Fe_3_O_4_ f-MNPs I–V, determined as a maximum magnetization characterized by parallel orientations of magnetic moments, varies from 45 to 70 emu g^−1^, as a result of nanoparticle size and surface oxidation (Figures 6A,B). These values are smaller than the respective value for a bulk Fe_3_O_4_, i.e., 89–92 emu g^−1^, and within agreement with various size Fe_3_O_4_ nanoparticles modified by different organic material. Generally, the value of *M*_s_ increases with increasing nanoparticle size (Herng et al., 2015). This remains in agreement with the results obtained by Rangam et al. (2017), where increasing *M*_s_ values are observed with increasing diameters obtained from XRD (Figure 6A). However, diamagnetic coating of nanoparticles causes decrease of *M*_s_ due to introducing surface spin disorder. Previously reported results provided evidences on modification of magnetic properties of Fe_3_O_4_ due to adsorption (Li et al., 2018), grain size (Liu et al., 2016), and functionalization (Soares et al., 2015). The oxidation of the surface interface of Fe_3_O_4_ MNPs I–V is reflected in increasing Fe 2p_3/2_ BE values and separation between Fe 2p_3/2_ octahedral 2+ component and plasmon loss of Fe 2p_3/2_ octahedral 2+ component (ΔFe_Oh_^2+^) (Figure 6B), justifying modification of magnetic properties resulting from slight modification of Fe_3_O_4_ stoichiometry and spin flipping at the interface of MNPs and functionalizing adsorbed molecules. The saturation magnetization increases in the following order: sample IV < sample II < sample III < sample V < sample I.

### Chemical State of Chlorine and Sulfur Contaminations

Chlorine was observed in Fe_3_O_4_ and samples I–V (0.1–1.9 at. %), and sulfur (1.1 at. %) was observed in sample IV (Supplementary Table S1). The chemical states of chlorine and sulfur contaminations were analyzed by fitting of Cl 2p (Figure 4, Supplementary Figure S5A, Supplementary Table S3) and S 2s (Supplementary Figure S5B) spectra. Chlorine chemical states are interpreted as follows: (i) [(CH_3_)_4_N]Cl and/or [N(C_2_H_5_)_4_]Cl (BE = 196.6 ± 0.3 eV), (ii) C(NH_2_)_3_Cl (BE = 198.2 ± 0.3 eV), and (iii) Met-Cl and/or (-CH_2_CHO(Cl)-)_n_ (BE = 199.9 ± 0.3 eV) (Wagner et al., 2012). Sulfur chemical state was interpreted as -SO_4_ (BE = 168.6 eV) (Wagner et al., 2012). The resulting BE values providing information on the chemical states of Cl and S indicate that ionic Cl and S are bonded to Fe_3_O_4_ MNPs and adsorbed functionalizing molecules, which may result from segregation due to applied temperature conditions (70°C-80°C) during functionalization of MNPs.

### Auger Parameters

**Figure 7:**
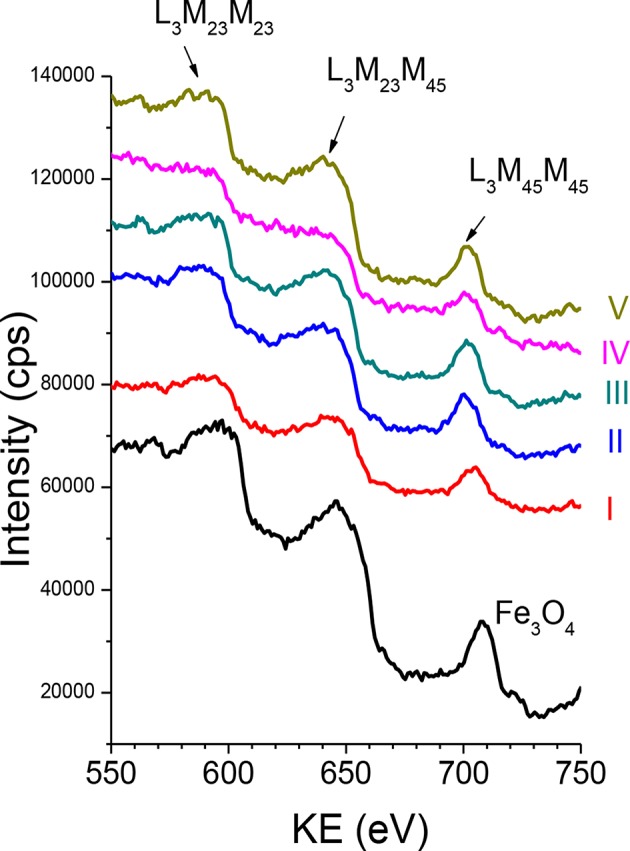
The Fe L_3_VV Auger spectra recorded from Fe_3_O_4_ MNPs and Fe_3_O_4_ f-MNPs I–V.

The Auger parameters are not sensitive to uniform charging of non-conductive specimens. Combining information resulting from photoelectron peak positions and peak positions of the Auger transitions (Figure 7), providing the Auger parameters, is a powerful tool for exploring the electronic structure of surfaces and interfaces in XPS-XAES studies.

The local electronic changes in charge redistribution and transfer at the atomic level can be investigated, analyzing the final state Auger parameter (Equation 1a) and the initial state Auger parameter (Equation 1b) defined as (Wagner, 1972; Gaarenstroom and Winograd, 1977):

(1a) $$\begin{array}{lll} \alpha & = & {\text{E}}_{\text{A}}+{\text{E}}_{\text{B}} \end{array}$$

(1b) $$\begin{array}{lll} \beta & = & {\text{E}}_{\text{A}}+3{\text{E}}_{\text{B},} \end{array}$$

where E_A_ is the kinetic energy (KE) of the Auger electron and E_B_ is the binding energy (BE) of the photoelectron. Changes in the Auger parameter between two environments due to the atomic potentials in the core of the atom and the core hole screening efficiency (extra-atomic relaxation), Δ*R* (Equation 2a), as well as between atomic potentials in the core of the atom at the atomic site, Δ*V* (Equation 2b) are defined as (Cole and Weightman, 1994; Cole et al., 1995):

(2a) $$\begin{array}{lll} \Delta \alpha & = & {\alpha_{Fe}}_{3O4}-\alpha_{env}=2\Delta R\; \end{array}$$

(2b) $$\begin{array}{lll} \Delta \beta & = & \beta_{Fe3O4}-\beta_{env}=2\Delta V, \end{array}$$

where α_*env*_ and β_*env*_ refer to functionalized Fe_3_O_4_ MNP surface. The final state effects (Δ*R*) refer to a shift in polarization energy at the core-ionized atom. This shift considers the charge transfer in a local valence band orbital of the core ionized atom and the contribution to the electronic relaxation energy of all the other atoms in the system. The initial state effects (Δ*V*) represent a chemical shift as a result of a ground state electronic structure and depend on bonding to neighboring atomic valence states. This shift is related to electronic states like, e.g., band structures, bond directionality, and structural parameters like atomic positions and Madelung constants of the bonded atom.

**Figure 8:**
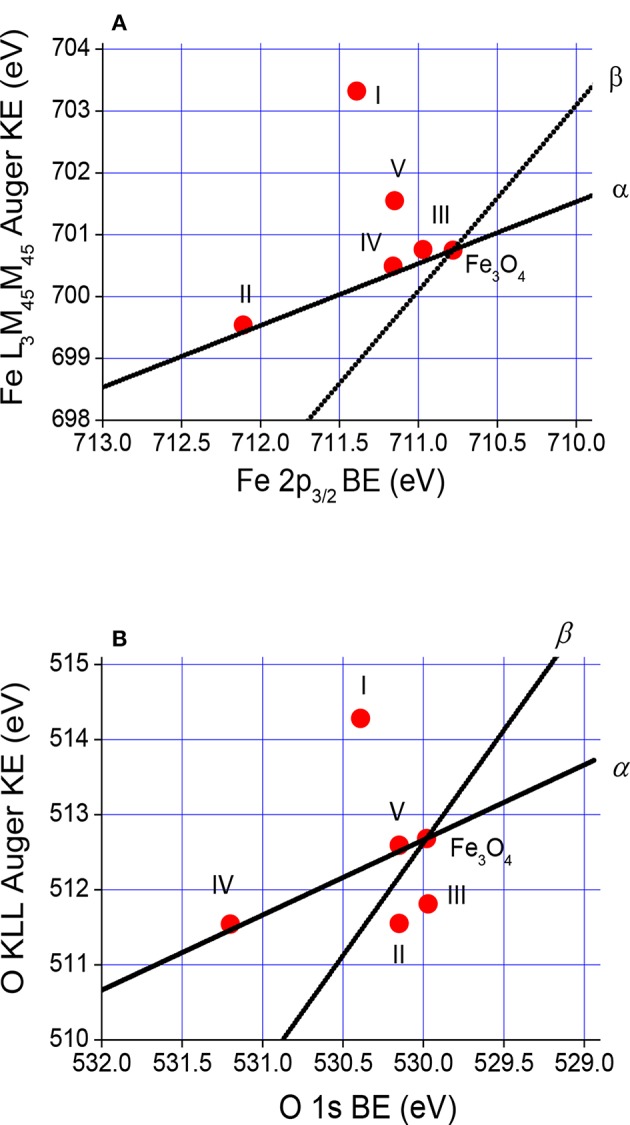
Wagner plots for **(A)** Fe L_3_M_45_M_45_ and Fe 2p_3/2_ spectra and **(B)** O KLL and O 1s spectra representing the investigated Fe_3_O_4_ MNPs and Fe_3_O_4_ f-MNPs I–V.

The Wagner plot for a given element and/or compound, known also as chemical state plot or chemical state diagram, displays in a compact form the values of photoelectron BE, Auger electron KE, and Auger parameters. Positions of compounds on this plot indicate relaxation shifts of various species and initial and final state effect contributions of various species. Therefore, Wagner plot information is related to the concept of ionicity, electronegativity, and polarizability. The Wagner plots for Auger Fe L_3_M_45_M_45_ electrons–Fe 2p_3/2_ photoelectron spectra and Auger O KLL electrons–O 1s photoelectron spectra representing the investigated Fe_3_O_4_ and Fe_3_O_4_ MNPs I–V samples are shown in Figure 8, whereas values of Auger spectra KE, BE of photoelectron spectra, and Auger parameters resulting from Equations 1a−2b are provided in Supplementary Tables S4A,B. It should be noted that Equations 2a and 2b are a good approximation in the case of core–core–core Auger transitions.

The spectral shape of Auger transition (Figure 7) is influenced by the valence band density of states and also by changes in the local density of states resulting from the screening of the initial core-hole. Therefore, any change of Auger spectral profile indicates modification of the local density of states. The core–core–core Auger electrons (L_3_M_23_M_2.3_) are not involved into bonding in contrast to core–core–valence Auger electrons (L_3_M_23_M_45_). The number of electrons from the Fe atom in the d states of the valence band, *n*^*d*^, evaluated from the ratio of core–core–valence to core–core–core Auger intensities (Allen et al., 1977) decreases in the following order: 0.92 (sample I), 0.89 (Fe_3_O_4_ MNPs), 0.86 (sample III), 0.85 (samples II and IV), and 0.83 (sample V), and exhibits no direct relation with cytotoxicity.

### Overlayer Thickness

The thickness of adsorbed molecule overlayers was evaluated using the following methods:

1. QUASES-Analyze (Tougaard, 1994–2002),
2. XPS MultiQuant (Mohai, 1999-2001, 2004), and
3. attenuation equation (Jablonski and Zemek, 2009)

and applying the inelastic mean free path (IMFP) values of photoelectrons from the G1 equation of Gries (1996).

QUASES-Analyze (Tougaard, 1994–2002) evaluates the surface morphology, i.e., type of depth profile, percent coverage and layer thickness from XPS spectra, and an inelastic background in the vicinity of the photoelectron peak. Exemplary results of QUASES-Analyze evaluations using the Buried Layer (BL) model without a standard and models of BL and Active Substrate (AS) with a Fe_3_O_4_ standard are shown in Supplementary Figures S6A–C, respectively. The values of surface coverage, adsorbed molecule overlayer thickness resulting from QUASES-Analyze BL and AS models without and with Fe_3_O_4_ standard, and averaged overlayer thickness are listed in Table 2.

**Table 2:** Coverage and overlayer thickness resulting from QUASES-Analyze Buried Layer (BL) model without a standard, and BL and Active Substrate (AS) models with a Fe_3_O_4_ standard.

**Sample**	**Analyze BL No standard thickness (nm)**	**Analyze BL Cov. (%)**	**Analyze BL Thickness (nm)**	**Analyze AS Thickness (nm)**	**Av. Thickness (nm)**
III	1.34	50.6	1.19	1.2	1.24 ± 0.08
I	1.5	42.2	1.6	1.58	1.56 ± 0.05
V	1.51	70.5	1.58	1.59	1.56 ± 0.04
IV	2.38	61.0	2.26	2.27	2.30 ± 0.07
II	1.38	58.9	1.5	1.5	1.46 ± 0.07

*Evaluation is performed from Fe 2p_3/2_ spectra recorded from Fe_3_O_4_ and Fe_3_O_4_ f-MNPs I–V. Sample f-MNPs I–V are listed in the order of decreasing cytotoxicity*.

The thickness resulting from XPS MultiQuant (Mohai, 1999-2001, 2004) was evaluated using the Layers-on-Sphere model. Although the particles are small, the large difference between the IMFP of the overlayer and a core allows using it (particle radius set to 10 nm). The composition, molecular weight, and IMFP values from G1 equation (Gries, 1996) for each adsorbed molecules are listed in Table 3. Other parameters applied are as follows: Al K_α_ excitation, Scofield cross section, and Reilman angular corrections for an analyzer input angle of 54.4°. The surface of Fe_3_O_4_ nanoparticles is oxygen deficient in comparison to functionalized nanoparticles I–V (Figure 3). After functionalization, the calculated thickness of contamination assuming CH_x_ hydrocarbon and possible other oxidized states (Table 1) is almost the same but the quantity of the missing oxygen is different. Due to the oxygen deficiency of the nanoparticles and their original carbonaceous contamination, the calculated layer thickness must be considered critically; presumably, the values calculated without oxygen are closer to reality. In the case of nitrogen-containing molecules, the nitrogen deficiency suggests that beside the adsorbed molecules, carbonaceous contamination is also present. It may be true for the other molecules as well. The values of overlayer thickness evaluated from XPS MultiQuant are listed in Table 3.

**Table 3:** Parameters for determining overlayer thickness from XPS MultiQuant (Mohai, 1999-2001, 2004), attenuation equation (Equation 3) (Jablonski and Zemek, 2009), IMFP values for various overlayers from Gries G1 equation (Gries, 1996), and comparison of overlayer thickness values resulting from XPS MultiQuant, attenuation equation and effective thickness from QUASES-Analyze for Fe_3_O_4_ MNPs and Fe_3_O_4_ f-MNPs I–V.

**Sample**	**Density (g cm^**−3**^)**	**Atomic weight (g mol^**−1**^)**	***N*_**v**_**	**Overlayers IMFP_**Gries**_ (nm)**	***t* (nm) Equation (3) (IMFP_**Gries**_)**	***t* (nm) XPS MultiQuant (IMFP_**Gries**_)**	**Effective Av. thickness QUASES-analyze (nm) (IMFP_**Gries**_)**
Fe_3_O_4_	5.18	231.533	–	1.55	–	–	–
III	1.90	90.03 (anhydrous)	34	2.20	1.0	1.11	0.63
I	1.56	118.09	46	2.06	0.74	0.68	0.66
V	1.4601	147.13	58	2.11	1.42	1.04	1.10
IV	1.665	192.12 (anhydrous)	74	2.16	1.22	–	1.40
II	1.43	174.20	70	2.00	1.37	0.66	0.86

*Sample f-MNPs I–V are listed in the order of decreasing cytotoxicity. The IMFP values were calculated for Fe 2p_3/2_ photoelectron kinetic energy*.

The following attenuation equation was applied (Jablonski and Zemek, 2009):

(3) $$\begin{array}{l} t=\lambda \cos\alpha \ln\;\left(R+1\right) \end{array}$$

Where λ is the IMFP from Gries G1 equation (Gries, 1996) and *R* is given by Equation 4:

(4) $$\begin{array}{l} R=\left(\frac{I_{i}^{l}}{I_{j}^{s}}\right)\left(\frac{I_{j}^{o}}{I_{i}^{\infty }}\right) \end{array}$$

Where $I_{i}^{\infty }$ is the intensity of the photoelectron signal from an infinitely thick layer, $I_{j}^{o}$ is the signal intensity from the uncovered substrate, $I_{i}^{l}$ is the intensity of the photoelectron signal from a layer of a thickness *t*, and $I_{j}^{s}$ is the intensity of photoelectron signal from a substrate covered by a layer of thickness *t*.

**Figure 9:**
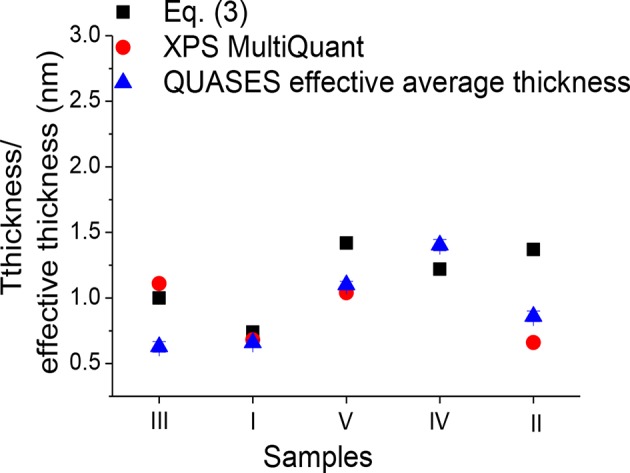
Comparison of overlayer thickness resulting from attenuation equation (Equation 3) (solid square), XPS MultiQuant (solid circle), and effective average thickness resulting from QUASES-Analyze (solid triangle) for Fe_3_O_4_ f-MNPs I–V. Sample f-MNPs I–V are shown in the order of decreasing cytotoxicity.

The $\frac{I_{i}^{\infty }}{I_{j}^{o}}$ can be measured in a separate experiment and/or calculated from Equation 5:

(5) $$\begin{array}{l} \frac{I_{i}^{\infty }}{I_{j}^{o}}=\frac{S\left(E_{i}\right)M^{l}\lambda_{i}^{l}{\left(d\sigma_{x}/d\Omega \right)}_{i}}{S\left(E_{j}\right)M^{s}\lambda_{j}^{s}{\left(d\sigma_{x}/d\Omega \right)}_{i}} \end{array}$$

where *S* is the spectrometer function, *dσ*_*x*_/*dΩ* is a differential photoelectric cross section, *M* is atomic density of a given element (number of atoms in unit volume), $M=\frac{No\rho }{A}$, *N*_*o*_ is Avogadro number, $\lambda_{i}^{l}$ is the IMFP for photoelectrons emitted in a layer *l* and moving in a layer *l*, $\lambda_{j}^{s}$ is the IMFP for photoelectrons emitted in a substrate *s* and moving in a layer *l*, ρ is a density, *A* is atomic mass, and α is a detection angle with respect to the surface normal. The values of parameters for determining the layer thickness from Equation 3, IMFPs from Gries G1 equation (Gries, 1996), and the layer thickness resulting from Equation 3 are provided in Table 3. Comparison of adsorbed molecule layer thicknesses obtained from QUASES-Analyze (so-called effective layer thickness, i.e., layer thickness multiplied by a coverage), XPS MultiQuant, and attenuation equation (Equation 3), respectively, is provided in Table 3 and Figure 9.

#### REELS Spectra

**Figure 10:**
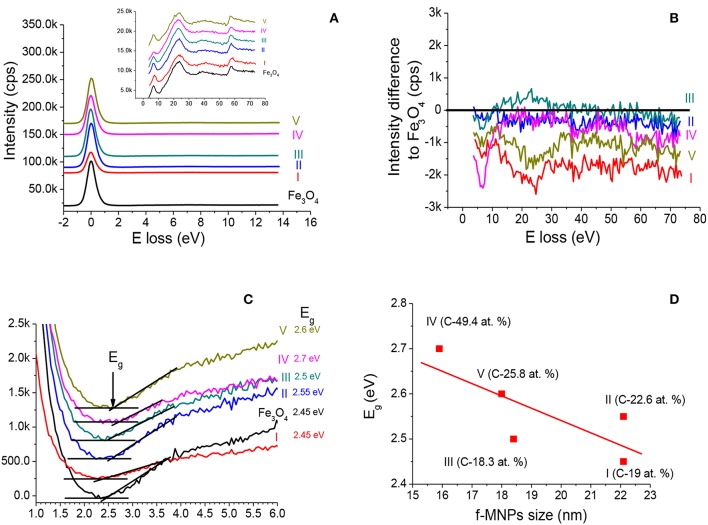
**(A)** Comparison of elastic peak and spectrum of inelastic losses of electrons on valence band electrons recorded from Fe_3_O_4_ MNPs and f-MNPs. **(B)** Differences between the spectrum of inelastic losses of electrons on valence band electrons recorded from Fe_3_O_4_ f-MNPs and the respective spectrum recorded from Fe_3_O_4_ MNPs. **(C)** Evaluation of band gap energy values from REELS spectra recorded from Fe_3_O_4_ MNPs and f-MNPs. **(D)** Dependence of band gap energy values on Fe_3_O_4_ f-MNPs nanoparticle size (Rangam et al., 2017) and surface carbon content (Supplementary Table S1).

Comparison of REELS spectra recorded from Fe_3_O_4_ MNPs and f-MNPs I–V and parameters derived from these spectra are shown in Figure 10. The electron scattering probabilities (elastic peak intensities) at 0 eV and inelastic scattering probabilities on valence band electrons in the region of about 80 eV above the elastic peak (electron inelastic losses) show variations due to functionalization (Figures 10A,B) with no remarkable changes in elastic peak full width at half maximum (FWHM) of 0.83 ± 0.02 eV. The presented changes (Figures 10A,B) reflect modification of surface electronic and optical properties. The quantitative analysis of REELS spectra combined with Tougaard QUEELS algorithm (Tougaard and Yubero, 2008) may provide detailed information on parameters of surface electronic and optical properties (Tougaard and Yubero, 2008; Tahir et al., 2018). The band gap energy (*E*_g_) value can be evaluated from REELS spectra since the plasmon loss peak exhibiting broad peaks with the energy in the vicinity of the elastic peak at 0 eV appears below the electron-hole interband transition. The onset of the loss spectrum is due to electron-hole excitation. The band gap energy was estimated from a linear fit line along the maximum negative slope at a point near the onset of the loss signal spectrum to the background level. The crossing of the linear fit line and the background level provides the band gap value (Figure 10C). The band gap energy values for Fe_3_O_4_ MNPs is 2.45 eV similarly as reported previously, i.e., 2.5 eV (Tahir et al., 2018). These values for Fe_3_O_4_ f-MNPs increase from 2.45 to 2.7 eV and exhibit decreasing dependence of band gap energy with decreasing carbon content and increasing nanoparticle size (Figure 10D). Similarly, increasing band gap energy values, i.e., from 2.4 to 2.9 eV, with increasing carbon content in Fe_3_O_4_ (Tahir et al., 2018) and decreasing Fe_3_O_4_ nanoparticle size (Kouotou et al., 2018) were reported previously. The inelastic scattering probability values showing changes due to functionalization (Figures 10A–D) provide evidences on modified optical and dielectric properties of the investigated surface (Tahir et al., 2018). No direct dependence of band gap energy of f-Fe_3_O_4_ MNPs on cytotoxicity tested for HeLa was observed, although this cytotoxicity was the highest for sample III of an intermediate nanoparticle size, the lowest carbon content, and the band gap energy value closest to that for Fe_3_O_4_.

#### Dependence of Cytotoxicity Tested for HeLa Cells on Surface Properties of Functionalized *Fe*_3_*O*_4_ Nanoparticles

Compilation of features indicating the differences in structural and chemical properties in the investigated surfaces Fe_3_O_4_ f-MNPs I–V in the order of decreasing cytotoxicity is compiled in Table 4. The highest cytotoxicity is observed for Fe_3_O_4_ f-MNPs with (i) the smaller surface coverage and thickness of biocompatible adsorbed molecules layers, (ii) the highest content of oxygen and carbon–oxygen functionalizing groups, (iii) the highest ratio of lattice O^2−^ and OH^−^ to C sp^2^ hybridizations on MNP surface, (iv) the highest ratio of adsorbed O^−^ and OH^−^ to C sp^2^ hybridizations due to adsorbed molecule layers, and (v) the closest electronic and optical properties to Fe_3_O_4_ shown in Auger parameters of XPS and Auger lines from Fe (Figure 8A) and REELS spectra (Figures 10A–D). No dependence of Cl and S contaminations and band gap energy was observed. No dependence of cytotoxicity on PDI and zeta potential values in the recorded range was shown. This would indicate that for the applied conditions of concentration, temperature, and pH, the sample homogeneity and ability for attachment to a negatively charged cell membrane are less important than the content of adsorbed molecule oxygen groups, which are responsible for generating ROS. Higher cytotoxicity is observed for MNPs of smaller hydrodynamic diameters (217.9–527.9 nm), indicating that adlayers of smaller polymerization degree will favor ROS generation.

**Table 4:** Dependence of cytotoxicity tested for HeLa cells on surface properties of Fe_3_O_4_ f-MNPs I–V.

**Sample**	**Av.% cell death**	**Cov. (%)**	**Av. *t* (nm)**	**C–OOH +C–OH C 1s (at. %)**	**Total O/C sp^**2**^ C 1s**	**Lattice O^**2−**^+OH^**−**^/C sp^**2**^**	**Ads (O^**−**^+OH^**−**^)_**ads**_ /C sp^**2**^**	***D*_**H**_ (nm)**	**PDI**	**Zeta potential (mV)**	**Overlayer interaction with MNPs**
Fe_3_O_4_	–	–	–	1.7	0.12	3.01	0.16	601.3	0.171	0.56	–
III	10.8	50.6	0.91	5.4	0.57	3.47	0.98	217.9	0.853	0.83	Weaker
I	10.7	42.2	0.69	4.3	0.34	3.27	0.48	527.9	0.101	−0.53	Stronger
V	9.2	70.5	1.19	3.2	0.29	2.77	0.56	256.7	0.246	0.28	Stronger
IV	7.5	61.0	1.31	3.1	0.10	0.57	0.35	717.7	0.039	0.19	Stronger
II	5.3	58.9	0.96	2.5	0.20	0.36	0.29	871.2	0.237	−0.23	Stronger

*Sample f-MNPs I–V are listed in the order of decreasing cytotoxicity. D_H_, hydrodynamic diameter; PDI, polydispersity*.

Although different iron oxides and also Fe_3_O_4_ have been previously applied for diagnosis and in tumor therapy (Sangaiya and Jayaprakash, 2018), the biocompatibility of functionalized Fe_3_O_4_ MNPs is competitive to Fe_3_O_4_ nanoparticles. The enhanced cytotoxicity for HeLa cells has been previously reported for L-cysteine-conjugated ruthenium oxy-hydroxide (RuO_x_(OH)_y_) in comparison to RuO_x_(OH)_y_ (Ganguly et al., 2018). This cytotoxicity of L-cysteine-conjugated RuO_x_(OH)_y_ increasing with the concentration of this agent was attributed to the selective ability of HeLa cells to create bonding with this surface. According to the above, the cytotoxicity of f-MNPs seems to be related to interaction of cells with the applied agent surface, where both oxygen groups, Fe_3_O_4_ lattice O^2−^ and OH^−^, and adsorbed O^−^ and OH^−^ play a role of adsorption and catalytic sites leading to the cytotoxicity of HeLa cells. Cytotoxicity was found to be higher for systems with a larger amount of double-carboxylic groups, which could enhance kinetics of Fenton reaction.

## Conclusions

Functionalization of Fe_3_O_4_ MNPs with different adsorbed molecules (samples I–V) to increase biocompatibility of Fe_3_O_4_ MNPs provides no modification in biocompatibility on L929 cells. However, it leads to variation in cytotoxicity on HeLa cells decreasing in the order III ≈ I > V > IV > II due to chemical and morphology modification of Fe_3_O_4_ MNPs.

The adsorbed layers provide f-MNPs of various physicochemical properties since adsorption of amino acids leads to modification of their surface and interface, providing nanoparticles of different hydrodynamic diameters, polydispersities, and zeta potentials. Functionalization provides adsorbed layers on Fe_3_O_4_ MNPs of various thicknesses and partial dissolution of oxalic, glutamic, and citric acids in nanoparticles. The presence of Fe_3_O_4_ MNPs and adsorbed layer of different thicknesses is confirmed by FTIR-S and UV-vis absorption spectra. The adlayer thickness values resulting from UV-vis and QUASES are in a reasonable agreement. The adsorbed layers have different degrees of polymerization confirmed by hydrodynamic diameter value. The adsorption behavior of amino acids on MNPs confirmed by FTIR-S results in weaker (oxalic acid) and/or stronger (succinic, L-arginine, citric, glutamic acids) interactions between adlayers and MNPs and different zeta potential values of nanoparticles. The C/O atomic content ratio is larger at the surface than in the bulk, indicating formation of functionalizing carbon–oxygen layers with oxygen deficiency in comparison to Fe_3_O_4_ MNPs. These carbon–oxygen layers show C sp^2^, C sp^3^, and carboxyl (C–OOH) groups and also C–N, C–${\text{NH}}_{3}^{+}$, C–NO_2_, and C–NO_3_ from adsorbed molecule layers present at the surface, whereas Fe_3_O_4_ MNPs and f-MNPs show the presence of lattice O^2−^ and OH^−^ and adsorbed O^−^ and OH^−^. The coverage of functionalizing adsorbed layers is 40–50% (oxalic and succinic acids) and 60–70% (L-arginine and citric and glutamic acid) and overlayer effective thickness is 0.69–1.31 nm. Such functionalization influences the magnetic, electronic, and optical properties of Fe_3_O_4_ MNPs. The modification of magnetic properties is manifested in changes of ratio of Fe 2p_3/2_ 2+ and 3+ tetrahedral and octahedral components and separation of Fe 2p_3/2_ photoelectron transition from inelastic plasmon. Modification of surface electronic charge redistribution and electronic and optical properties of f-MNPs is shown in the Auger parameters (derived from XPS and Auger spectra) and elastic/inelastic scattering probabilities of electrons on atoms and valence band electrons (derived from REELS spectra).

No dependence of cytotoxicity on polydispersity and zeta potential of Fe_3_O_4_ f-MNPs is observed, whereas the highest cytotoxicity is observed for f-MNPs with (i) a lower degree of polymerization, (ii) the smaller surface coverage and thickness of biocompatible adsorbed molecules layers, (iii) the highest content of oxygen and carbon–oxygen functionalizing groups, (iv) the highest ratio of lattice O^2−^ and OH^−^ to C sp^2^ hybridizations on MNP surface, (v) the highest ratio of adsorbed O^−^ and OH^−^ to C sp^2^ hybridizations on adsorbed molecule layers, and (vi) the closest electronic and optical properties to Fe_3_O_4_ shown in Auger parameters of XPS and Auger lines from Fe and REELS spectra. No dependence of Cl and S contaminations, band gap energy, and number of electrons from the Fe atom in the d states of the valence band was observed.

The enhancement of cytotoxicity of f-MNPs is related to interaction of cells with these surfaces, where both oxygen groups and increasing content of lattice O^2−^ and OH^−^, as well as adsorbed O^−^ and OH^−^ from biocompatible layers play a role of adsorption and catalytic sites and a large amount of double-carboxylic groups enhancing kinetics of Fenton reaction leading to cell damage. Since the cell viability and the type and mechanism of cell death are a more complex process, the results of the present work provide an indicative comparison of toxicity of the nanoparticles observed for HeLa cells focusing on nanoparticle surface properties and possible HeLa adsorption behavior.

## Data Availability Statement

All datasets generated for this study are included in the manuscript/Supplementary Files.

## Author Contributions

BL: XPS and REELS data evaluation, preparation of the manuscript. NR: XPS data evaluation, participating in preparation of the manuscript. PJ and IG: XPS data measurement, participating in preparation of the manuscript. JT: REELS data measurement, participating in preparation of the manuscript. LK: participating in preparation of the manuscript. MM: MultiQuant data evaluation, participating in preparation of the manuscript. PB: FTIR-S measurements and interpretation, participating in preparation of the manuscript.

### Conflict of Interest

The authors declare that the research was conducted in the absence of any commercial or financial relationships that could be construed as a potential conflict of interest.

## References

[B1] Allen G. C. Tucker P. M. Wild R. K. (1977). High resolution LMM Auger electron spectra of some first row transition elements. Surf. Sci. 68, 469–469. 10.1016/0039-6028(77)90240-0

[B2] Asgari S. Fakhari Z. Berijani S. (2014). Synthesis and characterization of Fe_3_O_4_ magnetic nanoparticles coated with carboxymethyl chitosan grafted sodium methacrylate. Nanostructures 4, 55–63. 10.7508/JNS.2014.01.007

[B3] Bahadur A. Saeed A. Shoaib M. Iqbal S. Bashir M. I. Waqas M. (2017). Eco-friendly synthesis of magnetite (Fe_3_O_4_) nanoparticles with tunable size: dielectric, magnetic, thermal and optical studies. Mater. Chem. Phys. 198, 229–235. 10.1016/j.matchemphys.2017.05.061

[B4] Bian J. Wang Y. Zhang Q. Fang X. Feng J. Li C. (2017). Fatty acid decarboxylation reaction kinetics and pathway of co-conversion with amino acids on supported iron oxide catalysts. RSC Adv. 7, 47279–47287. 10.1039/C7RA08507A

[B5] Bichara L. C. Lanús H. E. Ferrer E. G. Gramajo M. B. Brandán S. A. (2011). Vibrational study and force field pf the citric acid dimer based on the SQM methodology. Adv. Phys. Chem. 2011:347072 10.1155/2011/347072

[B6] Bordbar A. K. Rastegari A. A. Amiri R. Ranjbakhsh E. Abbasi M. Khosropour A. R. (2014). Characterization of modified magnetite nanoparticles for albumin immobilization. Biotechnol. Res. Int. 2014:705068. 10.1155/2014/705068 24963410 PMC4054909

[B7] Butenko Y. V. Krishnamurthy S. Chakraborty A. K. Kuznetsov V. L. Dhanak V. R. Hunt M. C. (2005). Photoemission study of onion like carbons produced by annealing nanodiamonds. *Phys. Rev*. B 71, 75420–75410. 10.1103/PhysRevB.71.075420

[B8] Cole R. J. Brooks N. J. Weightman P. Matthew J. A. D. (1995). Onset of d screening in alkali and alkaline earths. Phys. Rev. B 52, 2976–2982. 10.1103/PhysRevB.52.2976 9981370

[B9] Cole R. J. Weightman P. (1994). Separating ground state and screening contributions to chemical shifts. J. Phys. Condens. Matter 6, 5783–5790. 10.1088/0953-8984/6/29/020

[B10] Eltouny N. Ariya P. A. (2014). Competing reactions of selected atmospheric gases on Fe_3_O_4_ nanoparticles surfaces. Phys. Chem. Chem. Phys. 6, 23056–23066. 10.1039/C4CP02379J 25247461

[B11] Fujimoto A. Yamada Y. Koinuma M. Sata S. (2016). Origins of sp^3^C peaks in C_1s_ X-ray photoelectron spectra of carbon materials. Anal. Chem. 88, 6110–6114. 10.1021/acs.analchem.6b01327 27264720

[B12] Gaarenstroom D. W. Winograd N. (1977). Initial and final state effects in the ESCA spectra of cadmium and silver oxides. J. Chem. Phys. 67, 3500–3506. 10.1063/1.435347

[B13] Ganguly B. N. Maity B. Maity T. K. Manna J. Roy M. Mukherjee M.. (2018). L-cysteine-conjugated ruthenium hydrous oxide nanomaterials with anticancer active application. Langmuir 34, 1447–1456. 10.1021/acs.langmuir.7b01408 29281292

[B14] Gries W. H. (1996). An universal predictive formula for the inelastic mean free pathlengths of x-ray photoelectrons and Auger electrons. Surf. Interface Anal. 24, 38–50.

[B15] Grosvenor A. P. Cobe B. A. McIntyre N. S. (2004a). Examination of the oxidation of iron by oxygen using X-ray photoelectron spectroscopy and QUASES. Surf. Sci. 565, 151–162. 10.1016/j.susc.2004.06.210

[B16] Grosvenor A. P. Cobe B. A. McIntyre N. S. (2004b). Studies of the oxidation of iron by water vapour using X-ray photoelectron spectroscopy and QUASES. Surf. Sci. 572, 217–227. 10.1016/j.susc.2004.08.035

[B17] Grosvenor A. P. Kobe B. A. Biesinger M. C. Mcintyre N. S. (2004c). Investigation od multiplet splitting of Fe 2p XPS spectra and bonding in iron compounds. Surf. Interface Anal. 36, 1564–1574. 10.1002/sia.1984

[B18] Gupta A. K. Gupta M. (2005). Cytotoxicity suppression and cellular uptake enhancement of surface modified magnetic nanoparticles. Biomaterials 26, 1565–1573. 10.1016/j.biomaterials.2004.05.022 15522758

[B19] Herng T. S. Xiao W. Poh S. M. He F. Sutarto R. Zhu X. (2015). Achieving a high magnetization in sub-nanostructured magnetite films by spin-flipping of tetrahedral Fe^3+^ cations. Nano Res. 8, 2935–2945. 10.1007/s12274-015-0798-7

[B20] Hu Y. Liu W. Wu F. (2017). Novel multi-responsive polymer magnetic microgels with folate or methyltetrahydrofolate. RSC Adv. 7, 10333–10344. 10.1039/C6RA27114F

[B21] Infrared Spectroscopy-MSU Chemistry (2013). Available online at: http://www2.chemistry.msu.edu/faculty/reusch/virttxtjml/Spectrpy/InfraRed/infrared.htm (accessed July 31, 2019).

[B22] Jablonski A. Zemek J. (2009). Overlayer thickness determination by XPS using the multiline approach. Surf. Interface Anal. 41, 193–204. 10.1002/sia.3005

[B23] Kim D.-H. Kim K.-N. Kim K.-M. Lee Y.-K. (2009). Targeting to carcinoma cells with chitosan- and starch-coated magnetic nanoparticles for magnetic hyperthermia. J. Biomater. Res. A 88, 1–11. 10.1002/jbm.a.31775 18257079

[B24] Kim J. Jung J. Lee J. Na K. Park S. Hyun J. (2010). Amphiphilic comblike polymers enhance the colloidal stability of Fe_3_O_4_ nanoparticles. Colloids Surf. B Biointerfaces 76, 236–240. 10.1016/j.colsurfb.2009.10.042 19939645

[B25] Kouotou P. M. El-Kasmi A. Wu L. N. Wagas M. Tian Z. Y. (2018). Particle size-band gap energy–catalytic properties relationship of PSE-CVD-derived Fe_3_O_4_ thin films. J. Taiwan Inst. Chem. Engineers 93, 427–435. 10.1016/j.jtice.2018.08.014

[B26] Kövér L. Varga D. Cserny I. Tóth J. Tokési J. (1992). Some applications of high-energy, high-resolution Auger-electron spectroscopy using Bremsstrahlung radiation. Surf. Interface Anal. 19, 9–15. 10.1002/sia.740190106

[B27] Krishnan S. Raj J. C. Robert R. Ramanand A. Das J. S. (2007). Growth and characterization of succinic acid single crystals. Cryst. Res. Technol. 42, 1087–1090. 10.1002/crat.200710981

[B28] Kumar S. Rai S. B. (2010). Spectroscopic studies of L-arginine molecule. Indian J. Pure Appl. Physics 48, 251–255. Available online at: http://nopr.niscair.res.in/handle/123456789/7643

[B29] Kwok R. W. M. (2000). XPS Peak Fitting Program for WIN95/98 XPSPEAK, ver. 4.1. Shatin: Department of Chemistry, The Chinese University of Hong Kong.

[B30] Lesiak B. Kövér L. Tóth J. Zemek L. Jiricek P. Kromka A. (2018). C sp^2^/sp^3^ hybridisations in carbon nanomaterials—XPS and (X)AES study. Appl. Surf. Sci. 452, 223–231. 10.1016/j.apsusc.2018.04.269

[B31] Lesiak B. Zemek J. Jiricek P. Stobinski L. (2009). Temperature modification of oxidized multiwall carbon nanotubes studied by electron spectroscopy methods. Phys. Status Solidi B 246, 2645–2649. 10.1002/pssb.200982268

[B32] Li Z. Y. Jibran M. Sun X. Pratt A. Wang B. Yamauchi Y.. (2018). Enhancement of the spin polarization of an Fe_3_O_4_(100) surface by nitric oxide adsorption. Phys. Chem. Chem. Phys. 20, 15871–15875. 10.1039/C8CP02361A 29845166

[B33] Linh P. H. Chien N. V. Dung D. D. Nam P. H. Hoa D. T. Anh N. T. N. (2018). Biocompatible nanoclusters of O-carboxymethyl chitosan-coated Fe_3_O_4_ nanoparticles: synthesis, characterization and magnetic heating efficiency. J. Mater. Sci. 53, 8887–8900. 10.1007/s10853-018-2180-0

[B34] Liu Y. Cui T. Li Y. Zhao Y. Ye Y. Wu W. (2016). Effects of crystal size and sphere diameter on static magnetic and electromagnetic properties of monodisperse Fe_3_O_4_ microspheres. Mater. Chem. Phys. 173, 152–160. 10.1016/j.matchemphys.2016.01.053

[B35] Mahdavi M. Namvar F. Ahmad M. B. Mahamad R. (2013). Green biosynthesis and characterization of magnetic iron oxide (Fe_3_O_4_) nanoparticles using seaweed (*Sargassum muticum*). Molecules 18, 5954–5964. 10.3390/molecules18055954 23698048 PMC6270411

[B36] Massart R. (1981). Preparation of aqueous magnetic liquids in alkaline and acidic media. IEEE Trans. Magn. 17, 1247–1248. 10.1109/TMAG.1981.1061188

[B37] Mohai M. (1999-2001). Multimodel of X-ray photoelectron spectroscopy quantification program for 32-bit Windows, XPS MultiQuant, ver. 7.

[B38] Mohai M. (2004). XPS MultiQuant: multimodel XPS quantification software. Surf. Interface Anal. 36, 828–832. 10.1002/sia.1775

[B39] Muthuselvi C. Arunkumar A. Rajaperumal G (2016). Growth and characterization of oxalic acid doped with tryptophan crystal for antimicrobial activity. Der Chimica Sinica 7, 55–62. Available online at: https://www.researchgate.net/publication/319327894

[B40] Panias D. Taxiarchou M. Paspaliaris I. Kontopoulos A. (1996). Mechanisms of dissolution of iron oxides in aqueous oxalic acid solutions. Hydrometallurgy 42, 257–265. 10.1016/0304-386X(95)00104-O

[B41] Petran A. Radu T. Borodi G. Nan A. Suciu M. Turcu R. (2018). Effects of rare earth doping on multi-core iron oxide nanoparticles properties. Appl. Surf. Sci. 428, 492–499. 10.1016/j.apsusc.2017.09.160

[B42] Poulin S. França R. Moreau-Bélanger L. Sacher E. (2010). Confirmation of X-ray photoelectron spectroscopy peak attributions of nanoparticulate iron oxides, using symmetric peak component line shapes. J. Phys. Chem. C 114, 10711–10718. 10.1021/jp100964x

[B43] Rangam N. Sahu N. K. Jaiswal A. Jayesh B. (2017). Synthesis of surface grafted mesoporous magnetic nanoparticles for cancer therapy. J. Nanosci. Nanotech. 17, 5181–5188. 10.1166/jnn.2017.13853

[B44] Runowski M. Lis S. (2016). Synthesis, surface modification/decoration of luminescent-magnetic core/shell nanomaterials, based on the lanthanide doped fluorides (Fe_3_O_4_/SiO_2_/NH_2_/PAA/LnF_3_). J. Luminescence 170, 484–490. 10.1016/j.jlumin.2015.05.037

[B45] Sahu N. K. Gupta J. Bahadur D. (2015). PEGylated FePt-Fe_3_O_4_ composite nanoassemblies (CNAs): *in vitro* hyperthermia, drug delivery and generation of reactive oxygen species (ROS). Dalton Trans. 44, 9103–9113. 10.1039/C4DT03470H 25897960

[B46] Sangaiya P. Jayaprakash R. (2018). A review on iron oxide nanoparticles and their biomedical applications. J. Supercond. Novel Magn. 31, 3397–3413. 10.1007/s10948-018-4841-2

[B47] Scofield H. (1976). Hartree-Slater Subshell Photoionization Cross-sections at 1254 and 1487 eV. J. Electron Spectrosc. Relat. Phenom. 8, 129–137. 10.1016/0368-2048(76)80015-1

[B48] Sengupta P. K. Krimm S. (1985). Vibrational analysis of peptides, polypeptides, and proteins. Biopolymers 24, 1479–1491. 10.1002/bip.360240805 2412608

[B49] Shim S. H. Kim K. T. Lee Y. U. Jo W. H. (2012). Facile method to functionalize graphene oxide and its application to poly(ethylene terephthalate)/graphene composite. ACS Appl. Mater. Interfaces 4, 4184–4191. 10.1021/am300906z 22764836

[B50] Silverstein R. M. Bassler G. C. Morrill T. C. (1981). Spectrometric Identification of Organic Compounds. 4th ed. New York, NY: John Wiley and Sons. Available online at: http://www2.ups.edu/faculty/hanson/Spectroscopy/IR/IRfrequencies.html

[B51] Soares P. I. P. Lochte F. Echeverria C. Pereira L. C. J. Coutinho J. T. Ferreira I. M. M.. (2015). Thermal and magnetic properties of iron oxide colloids: Influence of surfactants. Nanotechnology 26:425704. 10.1088/0957-4484/26/42/425704 26421876

[B52] Stobinski L. Lesiak B. Zemek J. Jiricek P. (2012). Time dependent thermal treatment of oxidized MWCNTs studied by the electron and mass spectroscopy methods. Appl. Surf. Sci. 258, 7912–7917. 10.1016/j.apsusc.2012.04.127

[B53] Stobinski L. Lesiak B. Zemek J. Jiricek P. Biniak S. Trykowski G. (2010). Studies of oxidized multiwall carbon nanotubes in the temperature range from RT to 630 °C by the infrared and electron spectroscopy methods. J. Alloys Comp. 505, 379–384. 10.1016/j.jallcom.2010.05.185

[B54] Taghavi F. Saljooghi A. S. Gholizadeh M. Ramezani M. (2016). Deferasirox-coated iron oxide nanoparticles as a potential cytotoxic agent. Med. Chem. Commun. 7, 2290–2298. 10.1039/C6MD00293E

[B55] Tahir D. Ilvas S. Abdullah B. Armynah B. Kang H. J. (2018). Electronic properties of composite iron (II, III) oxide Fe_3_O_4_ carbonaceous absorber materials by electron spectroscopy. J. Electron Spectrosc. Rel. Phenom. 229, 47–51. 10.1016/j.elspec.2018.09.008

[B56] Tomitaka A. Yamaga T. Takemura Y. (2012). Magnetic nanoparticle hyperthermia using pluronic-coated Fe_3_O_4_ nanoparticles: an *in vitro* study. J. Nanomater. 2012:480626 10.1155/2012/480626

[B57] Tougaard S. (1994–2002). Software for Quantitative XPS/AES of Surface Nano-Structures by Analysis of the Peak Shape and Background, ver. 5.0. Odense: QUASES-Tougaard Inc.

[B58] Tougaard S. (1999–2001). Background Analysis of XPS/AES-QUASES Simple Backgrounds, ver. 2.2. Odense: QUASES-Tougaard Inc. Available online at: http://www.quases.com,.

[B59] Tougaard S. Yubero F. (2008). QUEELS-ε(k,ω)-REELS: Quantitative Analysis of Electron Energy Loss Spectra: Dielectric Function Determined by Reflection Electron Energy Loss Spectroscopy, ver. 3.0. Odense: QUASES-Tougaard Inc.

[B60] Wagner C. D. (1972). Auger lines in x-ray photoelectron spectrometry. Anal. Chem. 44, 967–973. 10.1021/ac60314a015 22309547

[B61] Wagner C. D. Naumkin A. V. Kraut-Vass A. Allison J. W. Powell C. J. Rumble J. R. Jr. (2012). NIST X-Ray Photoelectron Database, NIST SRD 20, ver. 4.1., online, PC. Gaithersburg: NIST, U.S. Department of Commerce.

[B62] Wei Y. Han B. Hu X. Lin Y. Wang X. Deng X. (2012). Synthesis of Fe_3_O_4_ nanoparticles and their magnetic properties. Proc. Engineering 27, 632–637. 10.1016/j.proeng.2011.12.498

[B63] Yamashita T. Hayes P. (2008). Analysis of XPS spectra of Fe^2+^ and Fe^3+^ ions in oxide materials. Appl. Surf. Sci. 254, 492–499. 10.1016/j.apsusc.2007.09.063

