# Supplementary material for: Surface Study of Fe3O4 Nanoparticles Functionalized With Biocompatible Adsorbed Molecules
Source: Front Chem. 2019 Oct 4;7:642. doi: 10.3389/fchem.2019.00642 (PMC6787174; doi:10.3389/fchem.2019.00642)
Supplement: Supplementary file 1 [file Data_Sheet_1.doc]

## Supplementary Material

**2 SUPPLEMENTARY FIGURES AND TABLES**

**2.1 SUPPLEMENTARY FIGURES**

## I II III

## succinic acid L-arginine oxalic acid

##
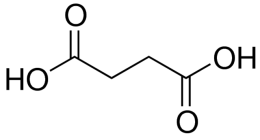

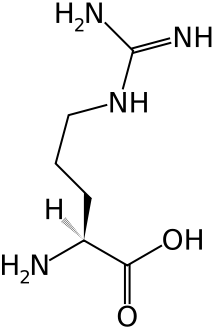

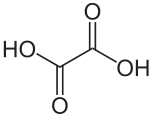


## IV V

## citric acid glutamic acid

##
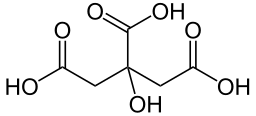

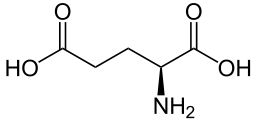


**SUPPLEMENTARY FIGURE S1 ** Structural formulae of adsorbed molecules functionalizing the Fe_3_O_4_ nanoparticles.


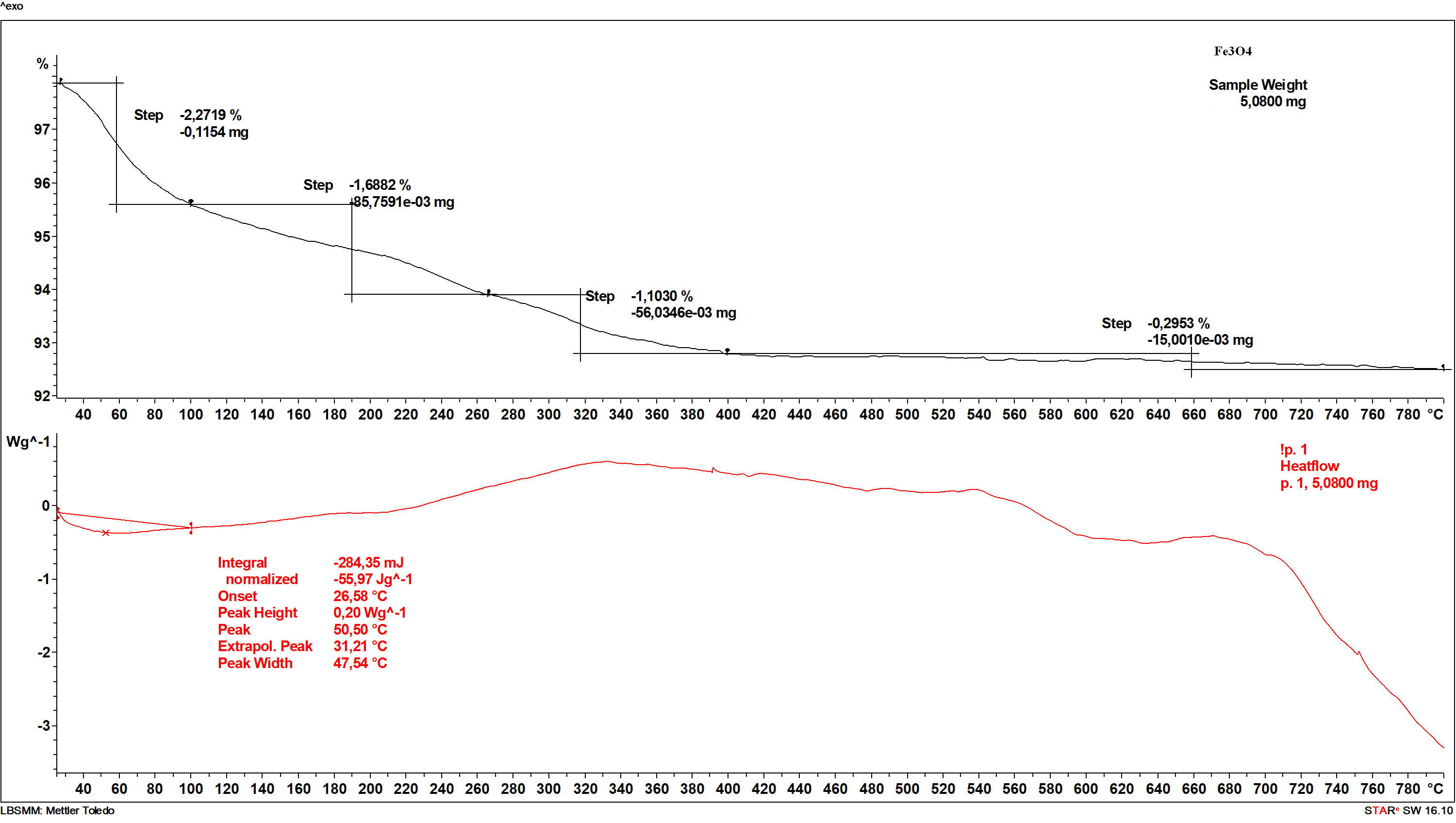


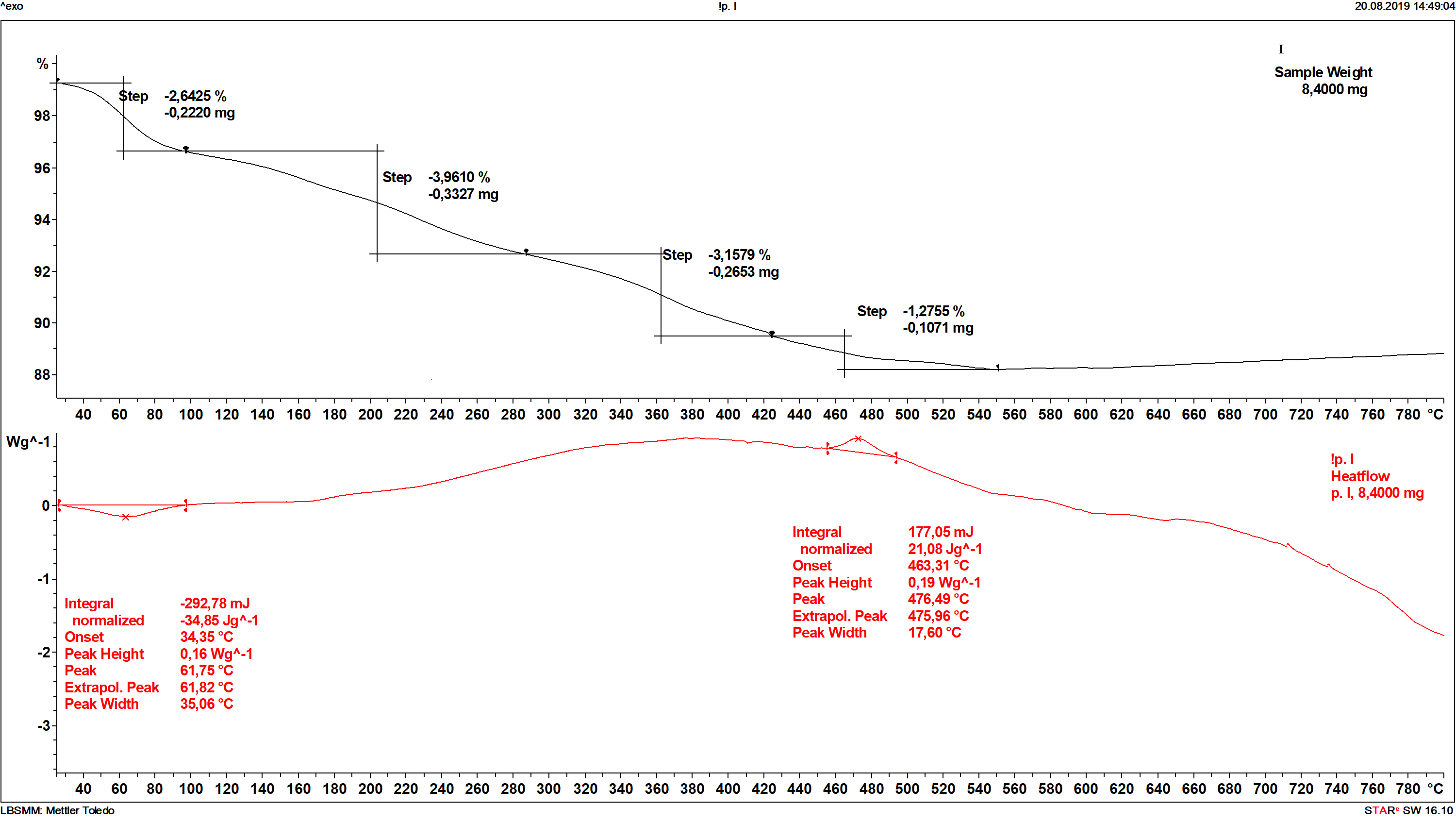


**SUPPLEMENTARY FIGURE S2A ** TGA and DSC thermoanalytical curves recorded from Fe_3_O_4_ MNPs and Fe_3_O_4_ f-MNPs I.


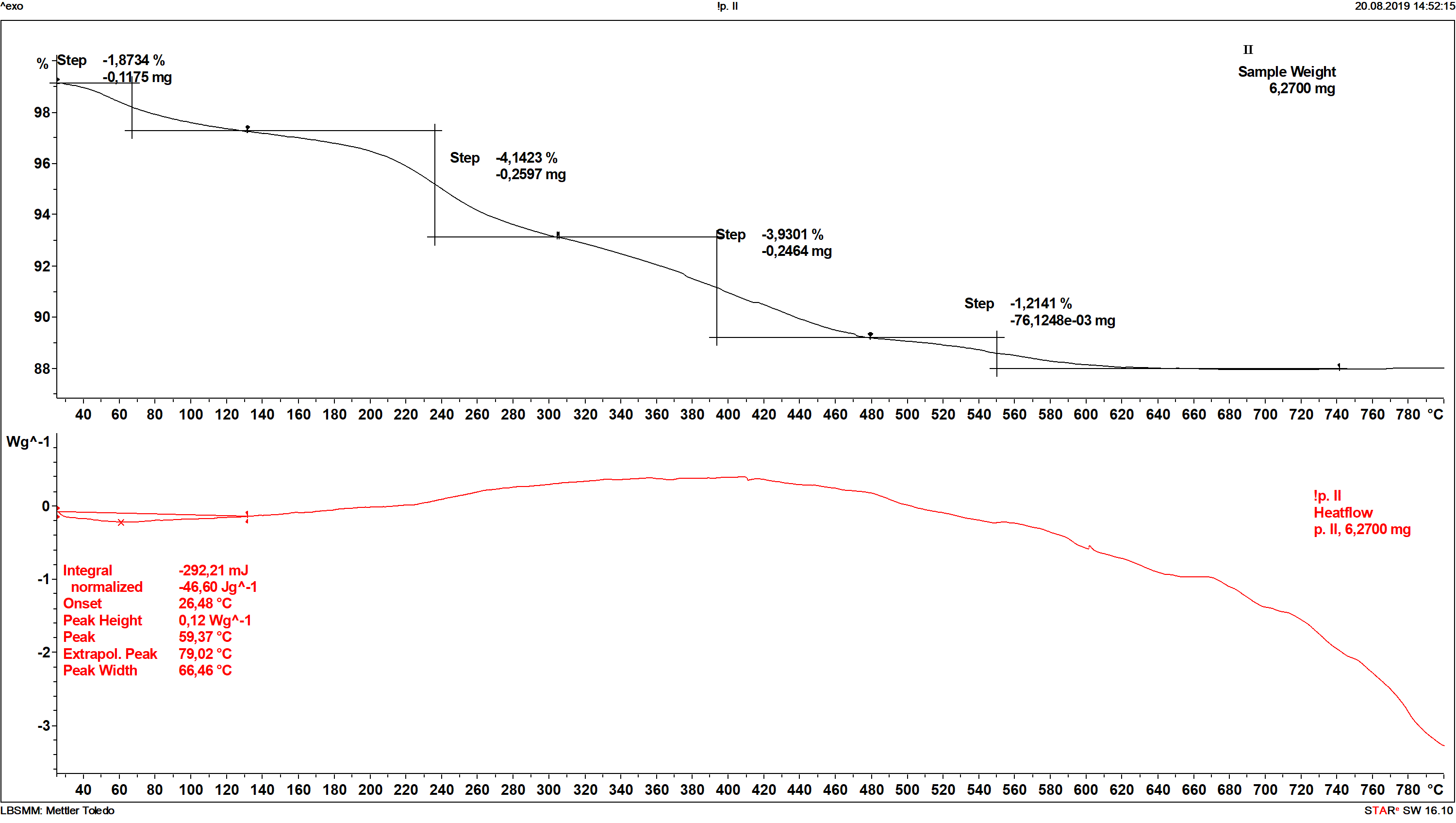


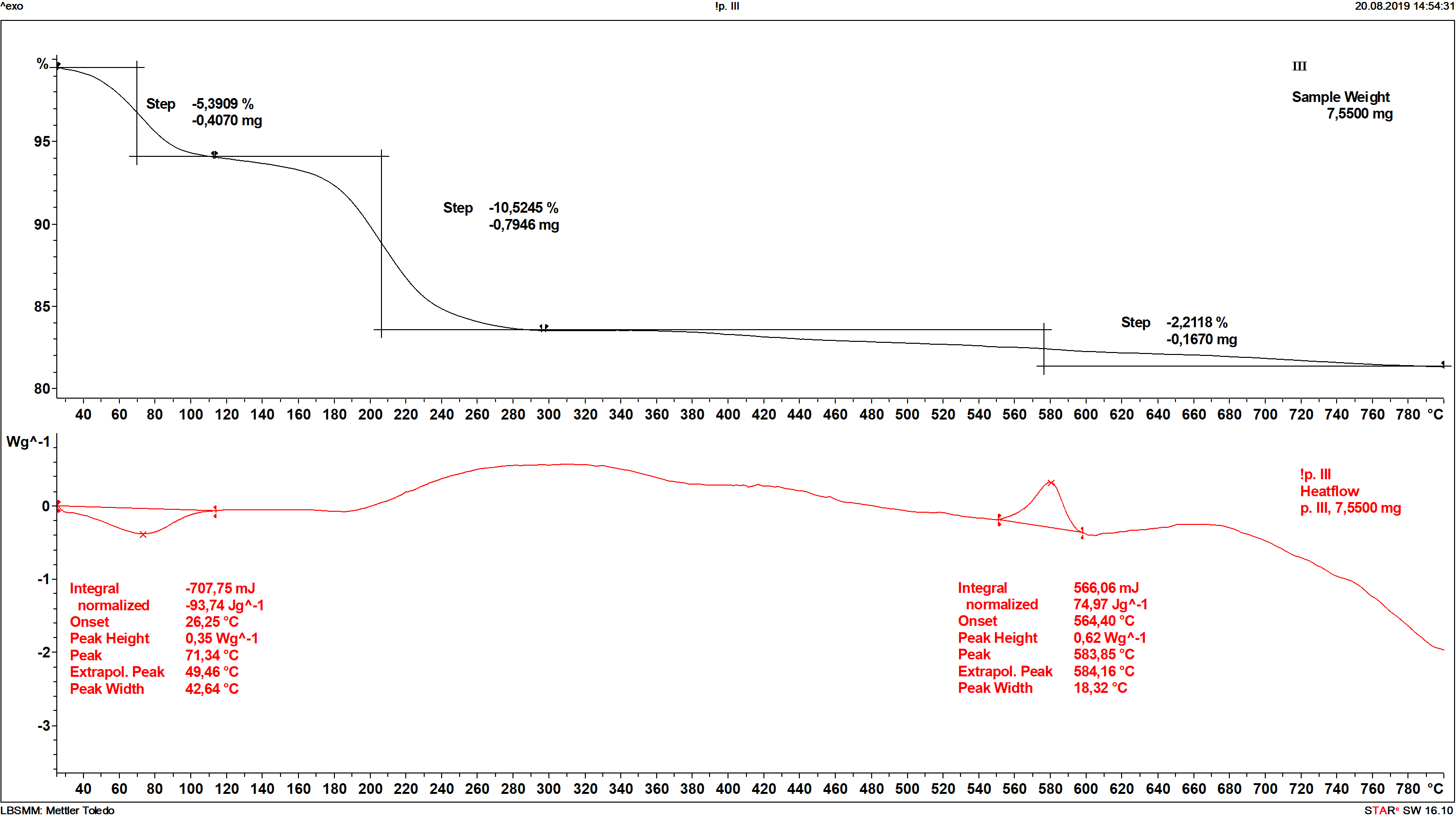


**SUPPLEMENTARY FIGURE S2B ** TGA and DSC thermoanalytical curves recorded from Fe_3_O_4_ f-MNPs II and III.


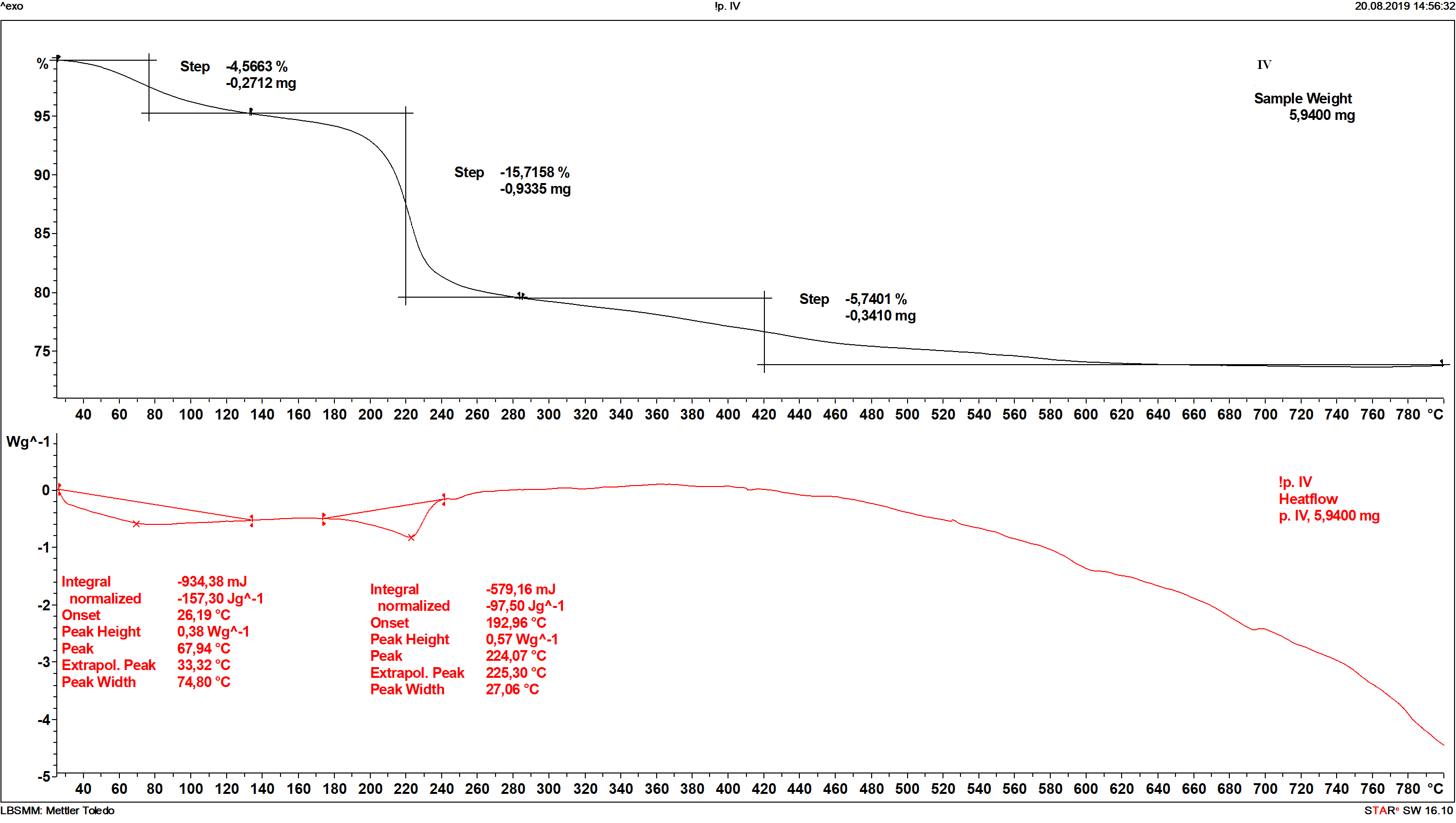


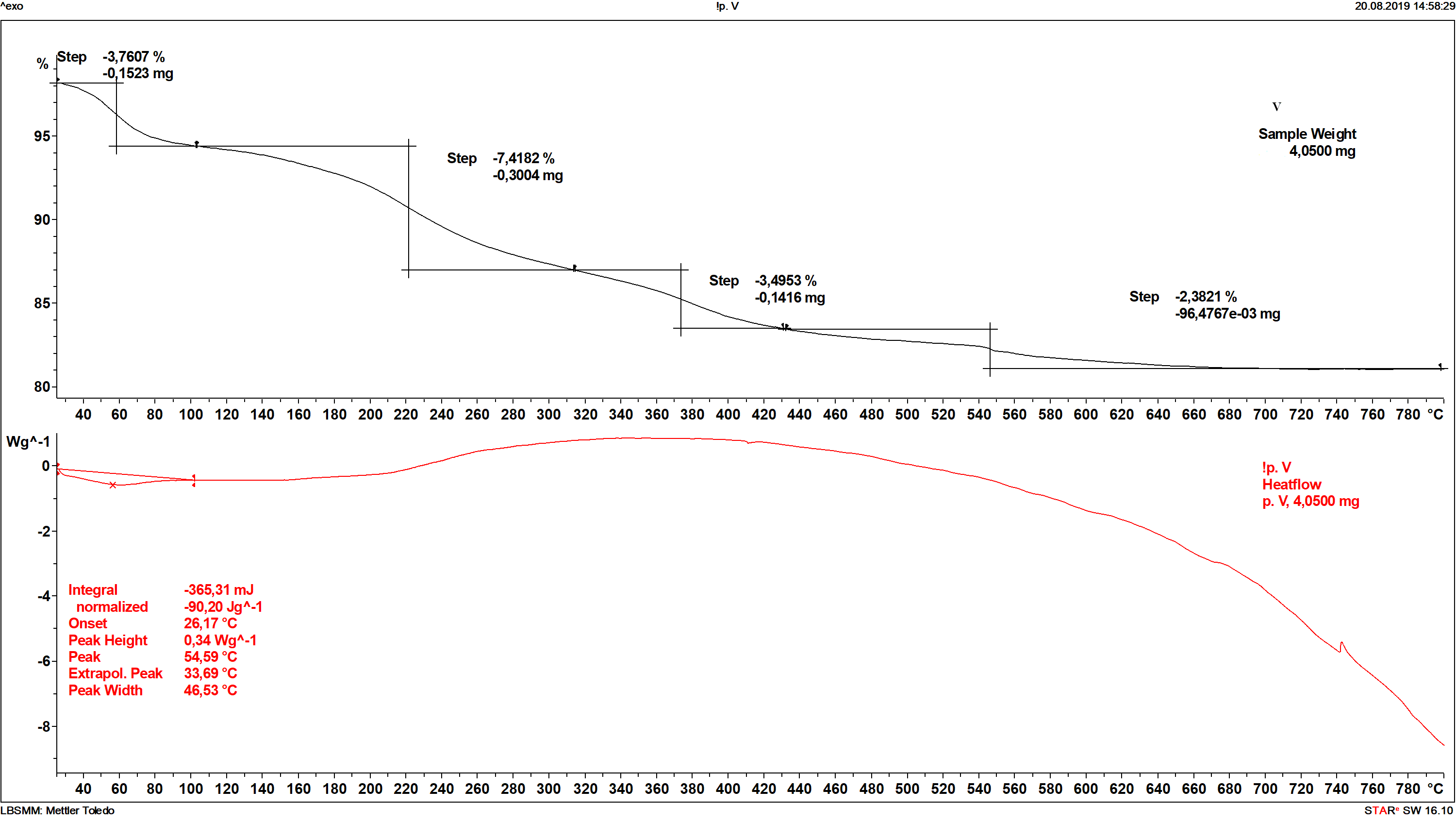


**SUPPLEMENTARY FIGURE S2C ** TGA and DSC thermoanalytical curves recorded from Fe_3_O_4_ f-MNPs IV and V.

EMBED Origin50.Graph


**SUPPLEMENTARY FIGURE S3A ** The XPS C 1s spectra recorded from Fe_3_O_4_ MNPs and Fe_3_O_4_ f-MNPs I-V fitted to different chemical forms.

**SUPPLEMENTARY FIGURE S3B ** The XPS O 1s spectra recorded from Fe_3_O_4_ and Fe_3_O_4_ f-MNPs I-V fitted to different chemical forms.

**SUPPLEMENTARY FIGURE S3C ** The XPS N 1s spectra recorded from samples II-V of Fe_3_O_4_ f-MNPs fitted to different chemical forms.

**SUPPLEMENTARY FIGURE S4 ** The XPS Fe 2p spectra recorded from Fe_3_O_4_ MNPs and Fe_3_O_4_ f‑MNPs I-V fitted to different chemical forms.

**SUPPLEMENTARY FIGURE S5A ** The XPS Cl 2p spectra recorded from Fe_3_O_4_ MNPs and Fe_3_O_4_ f-MNPs I-V fitted to different chemical forms.

**SUPPLEMENTARY FIGURE S5B ** The XPS S 2p spectrum recorded from IV Fe_3_O_4_ f-MNPs IV fitted to a chemical form.

**SUPPLEMENTARY FIGURE S6A ** Results of background subtraction of XPS Fe2p spectra recorded from various adsorbed molecule overlayers on Fe_3_O_4_ MNPs substrate (Fe_3_O_4_ f-MNPs I-V) using QUASES-Analyze Buried Layer (BL) without a standard.

**SUPPLEMENTARY FIGURE S6B ** Results of adjustment of XPS Fe2p spectra recorded from various adsorbed molecule overlayers on Fe_3_O_4_ MNPs substrate (Fe_3_O_4_ f-MNPs I-V) to respective spectra recorded from Fe_3_O_4_ standard using QUASES-Analyze Buried Layer (BL) model.

**SUPPLEMENTARY FIGURE S6C ** Results of adjustment of XPS Fe2p spectra recorded from various adsorbed molecule overlayers on Fe_3_O_4_ MNPs substrate (Fe_3_O_4_ f-MNPs I-V) to respective spectra recorded from Fe_3_O_4_ standard using QUASES-Analyze Active Substrate (AS) model.

**2.2 SUPPLEMENTARY TABLES**

# SUPPLEMENTARY TABLE S1 

# XPS quantitative results for Fe_3_O_4_ MNPs and Fe_3_O_4_ f-MNPs I-V. Samples f-MNPs I-V are listed in the order of decreasing cytotoxicity.

| **Sample** | **Elemental content (atomic %)** | | | | | |
| --- | --- | --- | --- | --- | --- | --- |
|  | **C** | **O** | **N** | **Cl** | **S** | **Fe** |
| Fe_3_O_4_ | 16.9 | 50.7 | - | 0.1 | - | 32.6 |
| III | 18.3 | 50.3 | 2.0 | 1.5 | - | 28.0 |
| I | 19.0 | 53.8 | - | 1.5 | - | 25.8 |
| V | 25.8 | 45.1 | 1.7 | 1.1 | - | 26.2 |
| IV | 49.4 | 35.1 | 2.4 | 0.4 | 1.1 | 11.6 |
| II | 22.6 | 45.9 | 2.2 | 1.9 | - | 27.3 |

### SUPPLEMENTARY TABLE S2A 

Atomic content and line positions (eV) of carbon-oxygen and carbon-nitrogen groups resulting from fitting of C 1s spectra for Fe_3_O_4_ MNPs and Fe_3_O_4_ f-MNPs I-V. Samples f-MNPs I-V are listed in the order of decreasing cytotoxicity.

| **Sample** | **C chemical states (atomic %)** | | | | | | |
| --- | --- | --- | --- | --- | --- | --- | --- |
|  | **C sp^2^** | **C sp^3^** | **C-OH** | **C=O** | **C-OOH** | **C-N  N=C-NH** | **C-NH(NH_2_)_2_^+^ C-NH_3_^+^ C-N=N** |
|  | **BE (eV)**  284.4 | **BE (eV)**  285.3 | **BE (eV)**  286.4 | **BE (eV)**  287.9 | **BE (eV)**  288.5±0.1 | **BE (eV)**  286.3±0.2 | **BE (eV)**  288.3±0.1 |
| Fe_3_O_4_ | 14.3 | 0.9 | 0.4 | - | 1.3 | - | - |
| III | 9.4 | 3.0 | 0.7 | - | 4.7 | 0.1 | 0.4 |
| I | 12.6 | 2.1 | - | - | 4.3 | - | - |
| V | 10.9 | 7.9 | - | - | 3.2 | 2.3 | 1.5 |
| IV | 31.0 | 8.4 | - | - | 3.1 | 2.3 | 4.6 |
| II | 12.7 | 6.4 | - | - | 2.5 | 1.0 | - |

**SUPPLEMENTARY TABLE S2B **

Atomic content and line positions (eV) of oxygen chemical forms resulting from fitting of O 1s spectra for Fe_3_O_4_ MNPs and Fe_3_O_4_ f-MNPs I-V. Samples f-MNPs I-V are listed in the order of decreasing cytotoxicity.

| **Sample** | **O chemical states (atomic %)** | | | | | |
| --- | --- | --- | --- | --- | --- | --- |
|  | **Lattice O^2-^**  **Fe_3_O_4_**  **FeOOH** | **Adsorbed O^-^_ads_** | **Lattice OH^-^ FeOOH** | **Adsorbed OH^-^_ads_** | **H_2_O** | **C-OOH** |
|  | **BE (eV)**  530.0±0.2 | **BE (eV)**  531.7±0.2 | **BE (eV)**  531.0±0.2 | **BE (eV)**  532.0±0.1 | **BE (eV)**  533.0±0.2  534.8±0.5  536.6±0.5 | **BE (eV)**  531.3±0.1  533.2±0.3 |
| Fe_3_O_4_ | 38.2 | - | 4.9 | 2.3 | 0.8; 1.0 ;0.9 | 1.3;1.3 |
| III | 31.0 | 5.1 | 1.6 | 4.1 | 0; 1.5; 1.0 | 3.0;3.0 |
| I | 35.7 | 3.0 | 5.5 | 3.0 | 0; 0; 0 | 3.3;3.3 |
| V | 20.6 | - | 9.6 | 6.1 | 0; 1.4; 0.8 | 3.3;3.3 |
| IV | 12.4 | 4.8 | 5.3 | 5.9 | 1.2; 0.1 | 2.7;2.7 |
| II | 28.1 | - | 7.7 | 3.7 | 1.0; 0.8 | 2.3;2.3 |

#### SUPPLEMENTARY TABLE S2C 

Atomic content and line positions (eV) of nitrogen chemical forms resulting from fitting of N 1s spectra for Fe_3_O_4_ MNPs and Fe_3_O_4_ f-MNPs I-V. Samples f-MNPs I-V are listed in the order of decreasing cytotoxicity.

| **Sample** | **N chemical states (atomic %)** | | |
| --- | --- | --- | --- |
|  | **C-N**  **N=C-NH** | **C-NH(NH_2_)_2_^+^**  **C-NH_3_^+^** | **C-NO_2_**  **C-NO_3_** |
|  | **BE (eV)**  399.7±0.3 | **BE (eV)**  401.6±0.2 | **BE (eV)**  404.7 |
| III | 0.3 | 1.7 | - |
| V | 1.0 | 0.6 | 0.1 |
| IV | 1.0 | 1.4 | - |
| II | 2.2 | - | - |

**SUPPLEMENTARY TABLE S3 **

Atomic content and line positions (eV) of chlorine chemical forms resulting from fitting of Cl 2p spectra for Fe_3_O_4_ MNPs and Fe_3_O_4_ f-MNPs I-V. Samples f-MNPs I-V are listed in the order of decreasing cytotoxicity.

| **Sample** | **Cl chemical state (atomic %)** | | |
| --- | --- | --- | --- |
|  | **[(CH_3_)_4_N]Cl**  **[N(C_2_H_5_)4]Cl** | **C(NH_2_)_3_Cl** | **Met-Cl**  **(-CH_2_CHO(Cl)-)_n_** |
|  | **BE (eV)**  196.6±0.3 | **BE (eV)**  198.2±0.3 | **BE (eV)**  199.9±0.3 |
| Fe_3_O_4_ | - | 0.06 | 0.04 |
| III | 0.1 | 1.0 | 0.4 |
| I | 0.5 | 0.7 | 0.3 |
| V | 0.1 | 0.6 | 0.4 |
| IV | 0.1 | 0.2 | 0.1 |
| II | 0.9 | 1.3 | 0.7 |

## SUPPLEMENTARY TABLE S4A 

Parameters of Fe L_3_M_45_M_45_ Auger and Fe 2p_3/2_ photoelectron spectra and Auger parameters for Fe_3_O_4_ MNPs and Fe_3_O_4_ f-MNPs I-V. Samples f-MNPs I-V are listed in the order of decreasing cytotoxicity.

| **Sample / Parameter** | **Fe_3_O_4_** | **III** | **I** | **V** | **IV** | **II** |
| --- | --- | --- | --- | --- | --- | --- |
| Fe L_3_M_45_M_45_ KE (eV) | 700.75 | 700.76 | 703.32 | 701.55 | 700.49 | 699.54 |
| Fe 2p_3/2_ BE (eV) | 710.78 | 710.97 | 711.39 | 711.15 | 711.16 | 712.11 |
| ΔE_A_ L_3_M_45_M_45_ | 0 | -0.01 | -2.57 | -0.8 | 0.26 | 1.21 |
| ΔE_B_ Fe 2p_3/2_ | 0 | -0.19 | -0.61 | -0.37 | -0.38 | -1.33 |
| *β* | 2833.1 | 2833.7 | 2837.5 | 2835.0 | 2834.0 | 2835.9 |
| *α* | 1411.5 | 1411.7 | 1414.7 | 1412.7 | 1411.7 | 1411.7 |
| Δ*β* | 0 | -0.6 | -4.4 | -1.9 | -0.9 | -2.8 |
| Δ*α* | 0 | -0.2 | -3.2 | -1.2 | -0.2 | -0.2 |

**SUPPLEMENTARY TABLE S4B **

Parameters of O KLL Auger and O 1s photoelectron spectra and Auger parameters for Fe_3_O_4_ MNPs and Fe_3_O_4_ f-MNPs I-V. Samples f-MNPs I-V are listed in the order of decreasing cytotoxicity.

| **Sample / Parameter** | **Fe_3_O_4_** | **III** | **I** | **V** | **IV** | **II** |
| --- | --- | --- | --- | --- | --- | --- |
| O KLL KE (eV) | 512.68 | 511.81 | 514.28 | 512.59 | 511.54 | 511.55 |
| O 1s BE (eV) | 529.98 | 529.97 | 530.39 | 530.15 | 531.20 | 530.15 |
| ΔE_A_ O KLL | 0 | 0.9 | -1.6 | 0.1 | 1.1 | 1.1 |
| ΔE_B_ O 1s | 0 | 0 | -0.4 | -0.2 | -1.2 | -0.2 |
| *β* | 2102.6 | 2101.7 | 2105.5 | 2103.0 | 2105.1 | 2102.0 |
| *α* | 1042.7 | 1041.8 | 1044.7 | 1042.7 | 1042.7 | 1041.7 |
| Δ*β* | 0 | 0.9 | -2.9 | -0.4 | -2.5 | 0.6 |
| Δ*α* | 0 | 0.9 | -2.0 | 0 | 0 | 1 |
